# Resveratrol alleviates oxidative stress induced by oxidized soybean oil and improves gut function via changing gut microbiota in weaned piglets

**DOI:** 10.1186/s40104-023-00851-2

**Published:** 2023-04-07

**Authors:** Yanan Gao, Qingwei Meng, Jianwei Qin, Qianqian Zhao, Baoming Shi

**Affiliations:** College of Animal Science and Technology, Northeast Agricultural University, No. 600 Changjiang Road, Xiangfang District, Harbin, China

**Keywords:** Inflammation, Intestinal barrier, Intestinal health, Oxidative stress, Oxidized soybean oil, Piglets, Resveratrol

## Abstract

**Background:**

Oxidized soybean oil (OSO) has been shown to impair growth and exacerbate inflammation, leading to intestinal barrier injury in animals. Recent evidence suggests important roles for resveratrol (RES) in the promoting growth performance, antioxidant capacity, anti-inflammatory, and regulate intestinal barriers in animals. Therefore, The objectives of this study are to investigate the effects of dietary RES (purity 98%) supplementation on the growth performance, antioxidant capacity, inflammatory state, and intestinal function of weaned piglets challenged with OSO.

**Methods:**

A total of 28 castrated weaned male piglets with a similar body weight of 10.19 ± 0.10 kg were randomly assigned to 4 dietary treatments for 28-d feeding trial with 7 replications per treatment and 1 piglet per replicate. Treatments were arranged as a 2 × 2 factorial with oil type [3% fresh soybean oil (FSO) vs. 3% OSO] and dietary RES (0 vs. 300 mg/kg).

**Results:**

The results showed that relative to the FSO group, OSO stress tended to decrease the average daily feed intake (ADFI), and decreased the activity levels of lipase, villus/crypt ratio (VCR), the mRNA expression of *FABP1*, *SOD2*, *IL-10* and *ZO-1* in the jejunum, and *SOD2*, *GPX1*, occludin and *ZO-1* in the colon, the levels of acetic acid in the colonic digesta, whereas up-regulated the mRNA expression of *IL-1β* and *TNF-α* in the jejunum (*P* < 0.05). Moreover, dietary supplementation with RES increased ether extract (EE), the activity levels of sucrase, lipase, α-amylase, villus height (VH) and VCR, the mRNA expression of *FABP1*, *SOD2*, *IL-10* and occludin in the jejunum, and *FABP1*, *PPAR-γ*, *GPX1*, occludin and *ZO-1* in the colon, and the abundance of Firmicutes, acetic and propionic acid, but decreased the levels of *D*-lactic acid in the plasma, the abundance of Bacteroidetes in the colonic digesta of weaned piglets compared to the non-RES group (*P* < 0.05). Meanwhile, in the interaction effect analysis, relative to the OSO group, dietary RES supplementation in the diets supplemented with OSO increased the activity levels of trypsin, VH in the jejunum, the abundance of Actinobacteria, the levels of butyric acid of weaned piglets, but failed to influence the activity levels of trypsin and VH, Actinobacteria abundance, the levels of butyric acid when diets were supplemented with FSO (interaction, *P* < 0.05). Relative to the OSO group, dietary RES supplementation in the diets supplemented with OSO decreased the activity levels of DAO in the plasma of weaned piglets but failed to influence the activity levels of DAO when diets were supplemented with FSO (interaction, *P* < 0.05). Relative to the FSO group, dietary RES supplementation in the diets supplemented with FSO decreased the level of propionic acid, whereas RES supplementation failed to influence the level of propionic acid when the diet was supplemented with OSO (interaction, *P* < 0.01).

**Conclusions:**

Inclusion of OSO intensified inflammatory states and impaired the intestinal health characteristics of weaned piglets. Dietary RES supplementation improved the antioxidant capacity, anti-inflammatory activity, and intestinal morphology. Further studies showed that the protective effects of RES on gut health could be linked to the decreased abundance of *Prevotella_1*, *Clostridium_sensu_stricto_6*, and *Prevotellaceae_UCG003* and increased levels of acetic and propionic acid.

**Supplementary Information:**

The online version contains supplementary material available at 10.1186/s40104-023-00851-2.

## Background

The energy values of lipids are much higher than those of grain, which can provide concentrated energy and essential fatty acids for mammals [1], slow the rate of feed through the digestive tract and give animals more time to digest and absorb other nutrients [2]. Soybean is a globally important oil crop, and soybean oil is considered an excellent source of polyunsaturated fatty acids (PUFAs), such as linoleic acid (LA, C18:2 n-6), alpha-linoleic acid (ALA, C18:3 n-3), and oleic acid (OA, C18:1 n-9), which participate in lipid metabolism, inflammatory response, and cholesterol synthesis [3]. However, PUFAs are more sensitive towards oxidation, especially when stored at high temperature and humidity [4]. After oxidation and rancidity, toxic secondary oxidation products will be produced, which will produce cytotoxicity and then induce changes in intestinal structure [5]. Furthermore, study has shown that impaired intestinal health causes decreased growth performance and severe diarrhea in animals exposed to thermally oxidized oil [6]. Oxidized soybean oil (OSO) containing high concentrations of lipid peroxidation products has been shown to impair growth and affect lipid metabolism characteristics in animals [7, 8]. Researchers have consistently demonstrated that the antioxidant statuses of weaned pigs [4, 9], broilers [7], and rats [10] that are fed oxidized oil are lower than those of animals that are fed nonoxidized oil [such as fresh linseed oil, fish oil, and fresh soybean oil (FSO)]. It has been stated that feeding animals OSO will exacerbate inflammation, leading to intestinal barrier injury [4, 9, 11]. A multitude of studies have shown that the gut microbiota plays an important role in suppressing pathogen infection and regulating nutrient digestion and absorption, the microbiota interacts with or even lives inside various niches within a mucosal barrier [12, 13]. Although, numerous studies have shown deleterious effects of exposure on animal gastrointestinal tracts, only a few studies have investigated the effects of oxidized oils on the intestinal microbial ecosystems of piglets [8, 9, 13, 14]. Furthermore, whether OSO induces intestinal damage and growth inhibition in weaned piglets by altering the gut microbiota remains unclear.

Accumulating evidences suggested that supplementation of additives with antioxidant function (such as short-chain fructo-oligosaccharides [13], pterostilbene [15], and polyphenols [16]) in the feed could alleviate the oxidative stress and improve the intestinal function of piglets. In recent years, several studies have explored the relationship between polyphenolic compounds and gut bacteria from a new research perspective. Resveratrol (RES; 3,5,4’-trihydroxystilbene) is one of the most abundant polyphenols in red grapes. Several studies reported that RES has a strong potential for promoting growth performance, antioxidative, anti-inflammatory, and regulate intestinal barriers in animals [17–20]. These beneficial effects of RES are largely associated with its physiological activities in the gastrointestinal tract [21]. It has been reported that RES promotes the proliferation of the *Lactobacillus* and *Bifidobacterium* in weaned piglets [20]. A previous study reported that piglets in the RES group show decreased abundance levels of bacteria in the genus *Ruminococcaceae UCG-005* and in the *Eubacterium coprostanoligenes* group relative to the diquat group, noteworthy, RES significantly increased the relative abundance of beneficial species, such as the genera *Clostridium *sensu stricto 1 and *Lachnospiraceae unclassified*, compared with the diquat and control groups [22]. Hence, we hypothesized that dietary RES can alleviate oxidative stress and improve gut health in OSO-challenged weaned piglets and that the effects may be related to changes in the gut microbiota. Hence, we hypothesize that dietary RES may alleviate oxidative stress and improve gut health in OSO-challenged weaned piglets and that the effects may be related to changes in the gut microbiota. In the present study, the effects of RES supplementation on the growth performance, intestinal oxidative stress, intestinal barrier integrity, and gut microbiota characteristics of weaned piglets challenged with OSO are investigated.

## Materials and methods

### Animals, diets, and management

The study was approved by the Animal Care and Use Committee of Northeast Agricultural University Institutional Animal Care and Use Committee (NEAU- [2011]-9). The animal experiment was conducted in the Acheng Experimental Base of Northeast Agricultural University. Twenty eight piglets [Duroc × (Landrace × Yorkshire)] were chosen from 7 litters on a large-scale breeding farm of the Gushi Agriculture and Animal Husbandry Group Co., Ltd. (Harbin, China), with 4 castrated weaned male piglets per litter. The selected piglets, which were weaned at 34.43 ± 0.31 days of age, had initial body weight (BW) of 10.19 ± 0.10 kg. Treatments were arranged as a 2 × 2 factorial design with oil type (3% FSO vs. 3% OSO) and dietary RES (0 vs. 300 mg/kg) as the factors. The dosage of RES adopted in the present study was based on our previous study [23]. RES were stored in light-proof containers, and fresh supplementary diets were prepared 50 kg each time. The FSO was purchased from Jiusan Grain and Oil Industry Group Co., Ltd. (Harbin, China). The oxidization degrees of soybean oil in different treatments were evaluated mainly by examining their peroxide values (POVs). Oil samples were analyzed according to the official methods of analysis of the American Oil Chemists’ Society (AOCS) for their peroxide values (AOCS Cd 8b-90) [24]. The OSO were continuously treated with FSO (POV = 11.7 mEqO_2_/kg) at 65 °C and bubbled air at rates of 4 L/min until the POVs reached approximately 384 mEqO_2_/kg. The POVs of OSO adopted in the present study were based on a previous study [25, 26]. The fresh and oxidized soybean oils were stored at −20 °C for further animal experiments. The nutrient and energy densities of the experimental diets were shown in Additional file 1: Table S1, and the nutrient levels of the diets were set to meet or exceed the nutritional requirements of swine based on the NRC (2012) [27]. All piglets were fed separately in a single stainless-steel metabolic cage (0.80 m × 0.75 m × 1.00 m) containing a water dispenser and feeding tank for free drinking and feeding. The feeding times were 6:30 and 17:30 every day, and the feeding amounts were 0.75 kg each time. Before feeding each morning, the remaining materials were collected and the weight was recorded. During the experiment, the pigpen temperature was maintained between 20 and 23 °C. Moreover, the relative humidity was maintained between 65% and 75%. The light regime was a 12-h light/12-h dark cycle. The piglets had no access to probiotics and/or antibiotics throughout the entire experiment.

### Quantification of long chain fatty acids

A 0.05-g FSO sample was taken into a test tube with plug, 2 mL n-hexane was added, and 2 mL 2 mol/L potassium oxide-methanol solution was added. Then, the tube shaken for 5 min and left for 30 min. The upper clarified solution was taken into a 10-mL centrifuge tube, and the minimum amount of anhydrous sodium sulfate was added. After 0.22 µm organic filtration membrane was passed, a sample solution of fatty acid methyl ester of l μL was absorbed with an injection needle. The fatty acid methylation of the OSO was the same as that of the FSO. All of the FAME were analyzed by using a gas chromatograph (Thermo Fisher Scientific, San Jose, CA, USA) equipped with a capillary column (100 m × 0.25 mm; 0.25 μm film thickness). The oven temperature of the gas chromatograph was held at 170 °C for 30 min, and then increased at a rate of 1.5 °C/min to 200 °C, 5 °C/min to 210 °C, and 15 °C/min to 250 °C for 1 min. The injector and flame-ionization detector temperatures were both set at 250 °C. Each FAME (1 μL) was injected into the split injection port (30:1 split ratio). The FAME were identified by comparing their retention times with an authentic standard. The ionization potential of the mass selective detector was 70 eV and the scan range was 50 to 550 *m*/*z*. Fat extractions were analyzed in duplicate to determine the percentage of lipids, which was reported as an average [28].

### Performance and diarrhea incidence

The body weight (BW) of piglets was evaluated on d 0 to 28 on the morning of the feed trial, and the weighing time was 5:30 every day. The daily feed intake of each pig during the experiment was recorded, the daily feed intake (ADFI), average daily gain (ADG), and average daily feed intake/average daily gain (F/G) were calculated. Clinical signs of diarrhoea were visually assessed each morning by three observers blinded to the treatments using a five-grade scoring system [29], which 1 = hard, 2 = slightly soft, 3 = soft, partially formed, 4 = loose, semi-liquid, 5 = watery, mucous like. Then, the average daily diarrhoea index per replication was calculated. Piglets with a index > 3 diarrhea between d 1 to 28 were identified as having diarrhea. Diarrhea index = sum of diarrhea index of each group of piglets during the test period/(number of test days × number of piglets per group) [30]. At the end of the experiment (d 24 to 27), all fresh feces without urine pollution were collected twice a day at 8:00 and 20:00, and other impurities were picked out, packed into plastic sealing bags, weighed and recorded [31]. To prevent the loss of fecal ammonia, after weighing the feces, 10 mL H_2_SO_4_ (10%, v/w) was added to the collected feces, and then the sample was stored in a −20 °C freezer.

### Sample collection and processing

After 28 days of the experiment, 10 mL of blood was collected from the ear vein of each weaned piglets fasted for 12 h with heparin vacuum anticoagulant tube. The plasma was obtained by centrifugation at 2000 × *g* for 20 min at 4 °C and stored at −20 °C until assayed. Then, the piglets were euthanized by electrocution and exsanguinated. After opening the abdominal cavity, the gastrointestinal tract was removed, and the jejunum and colonic intestinal segments were separated. Colonic digestate was collected from the tip of the colon into single sterile 2.0-mL frozen tubes, shock-frozen in liquid nitrogen and immediately stored at −80 °C for further experiments. Jejunal and colonic tissues that had been cleaned with ice-cold phosphate buffered solution (PBS) were collected using 2.0-mL cryogenic vials, shock-frozen in liquid nitrogen and immediately stored at −80 °C for further experiments. Two sections approximately 1.5 cm in length were carefully cut consecutively from the middle of the whole jejunum and colon and fixed in freshly frozen 4% paraformaldehyde. The sections were stored at 4 °C for morphological evaluation and histochemical staining.

### Determination of apparent nutrient digestibility

Before analysis, 4 d of faeces samples from each weaned piglets was thawed and evenly mixed. Approximately 200 g of the mixed fecal sample was removed and dried in an oven at 60 ± 5 °C for 48–72 h. Then, the dried fecal samples were crushed with a grinder and passed through a 40-mesh sieve for testing as follows. The nutrient digestibility coefficient was calculated using acid-insoluble ash (AIA) in feed and feces as exogenous indicator. The AIA content in diet and feces was determined according to (GB/T23742-2009) [32], and the concentration of hydrochloric acid was replaced with 4 mol/L. Dry matter (DM) was tested according to the national standard method for the determination of moisture and other volatile substances in feed (GB/T 6435-2006) [33]. Crude protein (CP) was tested according to the national standard method for the determination of crude protein in feed (GB/T 6432-1994) [34]. Crude fiber (CF) was tested according to the national standard filtration method for the determination of crude fiber content in feed (GB/T 6434-2006) [35]. Crude fat (EE) was tested according to the national standard method for the determination of crude fat in feed (GB/T 6433-2006) [36]. Samples of diets, feces and urine were analyzed for gross energy (GE) with an Isoperibol Oxygen Bomb Calorimeter (Parr 6400 Calorimeter, Moline, IL, USA). The calculation formula was as follows:

$$\mathrm{Digestibility}\;\mathrm{of}\;\mathrm a\;\mathrm{nutrient}\;\left(\%\right)=100-\left[100\times\frac{\left({\mathrm{CI}}_{\mathrm{input}}\times{\mathrm{CC}}_{\mathrm{output}}\right)}{\left({\mathrm{CI}}_{\mathrm{output}}\times{\mathrm{CC}}_{\mathrm{input}}\right)}\right]$$

In this equation, CI_input_ and CI_output_ are the concentration of index compound (AIA) in feed and feces, respectively; CC_input_ and CC_output_ are the concentration of component in feed and feces, respectively.

### Determination of digestive enzymes in the jejunum

The sample preparation processes and the jejunum digestive tissue enzyme activity levels were measured according to the manufacturer’s procedure. The activity of maltase in the jejunum was determined using a maltase assay kit (A082-3-1), the reagent was mixed with the sample and placed at 37 ℃ for 15 min, the reagent was placed in a colorimetric cup with a 1-cm optical diameter at the wavelength of 505 nm. The activity of sucrase in the jejunum was determined using a sucrase assay kit (A082-2-1), the reagent was mixed with the sample and placed at 37 ℃ for 15 min, the reagent was placed in a colorimetric cup with a 1-cm optical diameter at the wavelength of 505 nm. The activity of trypsin in the jejunum was determined using a trypsin assay kit (A080-2-1), the reagent was mixed with the sample and placed at 37 ℃ for 20 min, the reagent was placed in a colorimetric cup with a 0.5-cm optical diameter at the wavelength of 253 nm. The jejunum lactase activity was determined using a lactase assay kit (A082-1-1), the reagent was mixed with the sample and placed at 37 ℃ for 15 min, the reagent was placed in a colorimetric cup with a 1-cm optical diameter at the wavelength of 505 nm. The jejunum chymotrypsin activity was determined using a chymotrypsin assay kit (A080-3-1), the reagent was mixed with the sample and placed at 37 ℃ for 20 min, the reagent was placed in a colorimetric cup with a 1-cm optical diameter at the wavelength of 660 nm. The jejunum lipase activity was jejunum determined using a lipase assay kit (A054-1-1), the reagent was mixed with the sample and placed at 37 ℃ for 10 min, the reagent was placed in a colorimetric cup with a 1-cm optical diameter at the wavelength of 420 nm. α-Amylase in the jejunum was determined using an α-amylase assay kit (C016-1-1), the reagent was mixed with the sample, the reagent was placed in a colorimetric cup with a 1-cm optical diameter at the wavelength of 660 nm. All kits were set to zero and colorimetric with distilled water. All kits were from Nanjing Jiancheng Bioengineering Institute (Nanjing, Jiangsu, China).

### Plasma oxidative stress status and cytokines analyses

The following kits were used: total superoxide dismutase (T-SOD) assay kit (A001-1-1), the reagent was mixed with the sample and placed at room temperature for 10 min, the reagent was placed in a colorimetric cup with a 1-cm optical diameter at the wavelength of 550 nm, the distilled water was adjusted to zero and colorimetric. Glutathione peroxidase (GSH-Px) assay kit (A005-1-2), the reagent was mixed with the sample and placed at room temperature for 15 min, the reagent was placed in a colorimetric cup with a 1-cm optical diameter at the wavelength of 412 nm. Hydrogen peroxide (H_2_O_2_) assay kit (A064-1-1), the reagent was mixed with the sample, the reagent was placed in a colorimetric cup with a 1-cm optical diameter at the wavelength of 405 nm. Total antioxidant capacity (T-AOC) assay kit (A015-1-2), the reagent was mixed with the sample and placed at room temperature for 10 min at 35 °C, the reagent was placed in a colorimetric cup with a 1-cm optical diameter at the wavelength of 520 nm. Malondialdehyde (MDA) assay kit (A003-1-2), the reagent was placed in a colorimetric cup with a 1-cm optical diameter at the wavelength of 532 nm. All kits were set to zero and colorimetric with distilled water. The kits were all from Nanjing Jiancheng Bioengineering Institute (Nanjing, Jiangsu, China).

Other kits included the following: interleukin-1β ELISA kit (HY-H0001), the reagent was placed in a colorimetric cup with a 1-cm optical diameter at the wavelength of 450 nm. Interleukin-6 ELISA kit (HY-H0007), the reagent was placed in a colorimetric cup with a 1-cm optical diameter at the wavelength of 450 nm. Interleukin-8 ELISA kit (HY-H0008), the reagent was placed in a colorimetric cup with a 1-cm optical diameter at the wavelength of 450 nm. Tumor necrosis factor-α (TNF-α) ELISA kit (HY-H0019), the reagent was placed in a colorimetric cup with a 1-cm optical diameter at the wavelength of 450 nm. And porcine immunoglobulin A, G and M (IgA, IgG and IgM) (HY-759), the reagent was mixed with the sample and placed at 37 °C for 10 min, the measured wavelengths of IgA, IgG and IgM are 340 nm, 700 nm and 340 nm, respectively. All kits were set to zero and colorimetric with blank hole. The ELISA kits were all from Beijing Sino-UK Institute of Biological Technology (Beijing, China).

### Mucosal morphometry and epithelial proliferation in the jejunum and colon

For intestinal morphological analysis, after being fixed in paraformaldehyde solution at room temperature for 24 h, jejunal tissue specimens were dehydrated using a graded series of ethanol and xylene and then processed into paraffin blocks. A cross-section with a thickness of 5 μm was cut from each specimen and stained with hematoxylin (HHS32, Sigma-Aldrich, St. Louis, MO, USA) and eosin (318906, Sigma-Aldrich St. Louis, MO, USA). Three sections of jejunum and colon tissue were taken from each piglets, and selected typical field of view from each jejunum and colon tissue section, i.e., 3 with complete structures, to take photos. Nine villi and crypts were measured in each pig. Villus height (VH) and crypt depth (CD) were viewed on the light microscope. VH is the distance between the top of the villi and the midpoint of the connection between the villi of both crypts. CD is the distance between the midpoint of the connection between the villous junction of both crypts and the mucosal base [37]. Typical visual fields were selected from each jejunal tissue section, namely, three straight villi with complete structure and three complete crypts were measured. The assessor was blinded to the treatments using optical microscopy (Nikon Eclipse 80i Nikon, Tokyo, Japan) and NIS-Elements 3.0 Imaging Software. Data were analyzed using a digital microscope and photographed at 40 × magnification (BX53, Olympus, Tokyo, Japan). The adjacent jejunum and colon were fixed overnight in a 2.5% glutaraldehyde solution at 4 °C, and then these samples were treated for observation by electron microscopy. Epithelial proliferation was assessed using immunofluorescence in combination with a standardized quantification pipeline in the jejunum and colon mucosa. To determine the relative numbers of proliferative epithelial cells in the jejunum and colon mucosa samples, tissues were stained with the nuclear stain PCNA and Hoechst. (*n* = 3 randomly selected piglets from each treatment group).

### Immunofluorescence

(1)Prepreparation for paraffin section: The embedded wax blocks were frozen for 2 h and preheated in advance by an integrated machine, where they underwent spreading, baking and drying. Slices were sliced at 50 °C, baked at 80 °C for 30 min, and baked at 95 °C for 15 min. The sample slice was 4 μm. (2) The dewaxing hydration steps were ① xylene (I) 5 min; ② xylene (II) 5 min; ③ 100% ethanol twice, 5 min each; ④ the membrane of 3% H_2_O_2_ was broken for 10 min (1.8 mL methanol and 0.2 mL hydrogen peroxide were mixed well); ⑤ 95% ethanol 2 min; ⑥ 90% ethanol 2 min; ⑦ 85% ethanol 2 min; ⑧ 80% ethanol 2 min; and ⑨ 75% ethanol for 2 min. After completing the above steps, double distilled water wash 3 times, 5 min each. (3) The antigen repair steps included the sections being immersed in a repair box containing repair solution (4.75 mL antigen unmasking solution [H-3300, Vector, Shanghai, China] + 500 mL double steaming water) and boiled for 10 min in a microwave oven. The slices were then removed along with the antigen repair box and slowly cooled to room temperature. (4) For dyeing, each slide was added to 100 μL sealing solution (1 g Albumin Bovine V [PM11173, Biosharp, Shanghai, China] was dissolved in 100 mL PBS, stirred well, separated and set aside), pasted with sealing film. Incubate in a constant temperature and humidity incubator at 37℃ and 87% humidity for 30 min. Each slide was added to 100 μL monoclonal antibody zonula occludens-1 (ZO-1) [A0659, ABclonal, Wuhan, China], occludin [A2601, ABclonal], Ki67 [BSM-33070M, Bioss, Beijing, China]), pasted with sealing film, and incubated overnight at 4 ℃. The sample was placed at room temperature and rewarmed for 15 min. The antibody working liquid was removed and washed once with TBST buffer [0.001 L Tween-20 (1247, BioFroxx, Einhausen, Germany) was placed in 1 L TBS (BL600A, Biosharp, Guangzhou, China), stirred well, separated and set aside] for 5 min. Each slide was incubated with 100 μL fluorescent secondary antibody [goat anti-rabbit IgG (H + L) highly cross-adsorbed secondary antibody (A-11034, Thermo Fisher, Massachusetts, USA) goat anti-mouse IgG (H + L) highly cross-adsorbed secondary antibody (A-11029, Thermo Fisher)], pasted with sealing film, and incubated at room temperature for 1 h. The secondary antibody working liquid was removed, and the slide was washed with TBST buffer (1 L TBS buffer and 0.001 L Tween 20 solution were added to the buffer and mixed well). The cells were washed with TBS buffer solution 3 times for 5 min each time. (5) For nucleation, DAPI (BS097, Biosharp) working solution was added to the sample and incubated for 10 min at room temperature away from light. The DAPI working liquid was removed, and the sample was washed with TBST buffer once for 5 min and washed with TBS buffer 3 times for 5 min each time. (6) The anti-fluorescence attenuation tablet (MA0221, Meilunbio, Dalian, Liaoning Province, China) was added, and then the cover glass was affixed to seal the tablet. Then, the image was observed and collected under the fluorescence microscope. The diluent of primary antibody/secondary antibody was 1 g Albumin Bovine V dissolved in 100 mL PBS. The antibody dilution ratio is shown in Additional file 1: Table S2.

### Determination of diamine oxidase (DAO) activity and *D*-lactate contents in the plasma

Plasma DAO activity was determined by the DAO assay kit (A088-1-1), the reagent was mixed with the sample and placed at 37 ℃ for 10 min, the reagent was placed in a colorimetric cup with a 0.5-cm optical diameter at the wavelength of 340 nm, the distilled water was adjusted to zero and colorimetric. Plasma *D*-lactic acid content was determined by the *D*-lactic acid assay kit, (H263-1-1), gently shake the orifice plate, incubate at 37 °C for 15 min in the dark, and take readings at 450 nm with an enzyme label (M200pro, Männedorf, Switzerland). The kits were all from Nanjing Jiancheng Bioengineering Institute (Nanjing, Jiangsu, China). All procedures were performed in strict accordance with the manufacturer’s guidelines.

### Total RNA extraction, reverse transcription, and relative quantitative real-time PCR.

The reaction system and the thermal cycling conditions used for RT-PCR were adjusted according to our previous study [38]. An E.Z.N.A. Total RNA Kit I (R6834-01, Omega, Beijing, China) was used to extract the total RNA from the jejunal and colon mucosal samples according to the manufacturer’s instructions. The purity and concentration values of the total RNA samples were determined using a spectrophotometer to measure the absorbance values at 260 and 280 nm. The ratio of the optical density (OD) at 260 nm (OD_260_) to the OD_280_ ranged from 1.8 to 2.0. Then, the total RNA was reverse transcribed using a PrimeScript RT reagent kit (RR047A, TaKaRa, Dalian, China) according to the manufacturer’s directions. Subsequently, the cDNA was stored at −20 °C. All of the samples were distributed into 96-well plates, and every reaction was performed in triplicate. The primer sequences are shown in Additional file 1: Table S4. The average of β-actin and *GAPDH* was used as the internal control. The relative expression abundance of each target gene was calculated by the 2^−Δ Δ^^C^^t^ method, as previously described [39].

### Gut microbiota analysis

Bacterial genomic DNA was extracted from colonic digesta samples (Qiagen DNA Stool Mini Kit, 51504, Shanghai, China). DNA was quantified with a NanoDrop 2000 spectrophotometer (Thermo Scientific, Wilmington, NC, USA) and further assessed by running on 1% agarose gels. The V3-V4 hypervariable region of the 16S rRNA gene was amplified using specific primer pairs (forward 5’-ACTCCTACGGGAGGCAGCA-3’ and reverse 5’-GGACTACHVGGGTWTCTAAT-3’) with barcodes to construct the sequencing libraries (TruSeq® DNA PCR-Free Sample Prep Kit, Illumina, San Diego, CA, USA). The qualified DNA libraries were loaded in a NovaSeq platform with 2 × 250 bp paired-end sequencing. The paired-end reads were obtained and merged using FLASH software (V1.2.7, http://ccb.jhu.edu/software/FLASH/). Operational taxonomic units (OTUs) with 97% identity were gathered with Uparse (ver. 7.1, http://drive5.com/uparse/). Taxonomic annotation was performed using the Mothur algorithm (70% confidence) with the Silva Database (http://www.arb-silva.de/). The taxonomic composition of the bacterial community was then analysed. Through OTU clustering analysis, the abundance of OTUs in different samples can be obtained, and the microbial diversity in each sample can be evaluated, including the calculation and evaluation of the number (richness) of OTUs contained in samples and the stability (evenness) of community structure. According to the alignment results of each OTU representative sequence with the 16S rRNA database (RDP and NT-16S), the species classification statistics of OTUs were performed to obtain the species abundance of different taxonomic levels (phylum, family, genus and species).

### Quantification of short-chain fatty acids (SCFAs)

SCFAs, including acetic, propionic, butyric, isobutyric, isovaleric acids and valeric, were quantified by external standard methods using gas chromatography, and the preparation of protein-removed standard fluids and different concentrations of standard fluids was described in previous study [40]. Briefly, 1.5 g of colonic chyme samples was added to screw-capped tubes with 6 mL of distilled water. After mixing overnight at 4 °C and centrifugation at 1500 r/min for 10 min at 4 °C, 2 mL of supernatant from each sample was transferred to another centrifuge tube, and 400 µL of meta-phosphoric acid (25%, v/v) was added to remove the protein. The samples were then centrifuged at 12,000 r/min for 10 min at 4 °C. The resulting supernatants (1 mL each) were transferred into gas chromatography sample bottles and analysed using an Agilent 6890N GC (Palo Alto, CA, USA) coupled to a flame ionization detector with helium used as the carrier gas. An Agilent FFAP column [30 m × 0.53 mm i.d. × 1.00 µm (film thickness)] was installed for analysis, with a constant flow rate of 4.0 mL/min. The splitless injection volume was 0.2 µL of the sample. The injector and detector temperatures were 220 and 240 ℃, respectively. The GC oven temperature was held at 90 °C for 1 min and then increased to 190 °C at a rate of 20 °C/min and held for 3 min. Samples were run in triplicate, with a coefficient of variation less than 15% within triplicate samples 3 used for quality control.

### Statistical analysis

For data analysis, SPSS 23.0 statistical software was used to conduct a two-factor ANOVA on the test data. The statistical model included the main effects and interaction effects of OSO and RES. When the interaction is significant, Tukey HSD’s multiple comparisons were performed find the specific differences. Moreover, the Chi-square test was used to analyze diarrhea incidence. The statistical results are expressed as the mean ± standard error of the mean (SEM). *P* ≤ 0.05 was considered statistically significant, and 0.05 < *P* < 0.10 was considered a trend. RDA (Redundancy analysis) was used to determine the relationship between the intestinal flora and intestinal enzyme activities, antioxidant capacity, inflammatory factors, intestinal histomorphology, short-chain fatty acids. Pearson correlation analysis was used to explore the significant correlation between the intestinal microbiota and antioxidant indices, inflammatory factors, intestinal histomorphology, intestinal enzyme activities, and short-chain fatty acids.

## Results

### Growth performance and nutrient digestibility

As revealed in Table 1, it was found that relative to the FSO group, the inclusion of OSO tended to decrease the ADFI (*P* = 0.060). Inclusions of OSO or RES in diets failed to influence the ADG, F/G, and final weight (FW) values of weaned piglets. Furthermore, we evaluated the apparent digestibility levels of weaned piglets (Table 2). Relative to the non-RES group, dietary RES improved EE (*P* < 0.05). There was no difference in DM, CP, GE, CF, and the diarrhea index between groups over the 28 d period (*P* > 0.05, Table 3).

**Table 1:** Effects of resveratrol (RES) supplementation on growth performance in oxidized soybean oil (OSO)-challenged weaned piglets^1^

Item^2^	Fresh soybean oil		Oxidized soybean oil		SEM	*P*-value		
	0 mg/kg RES	300 mg/kg RES	0 mg/kg RES	300 mg/kg RES		Oil	RES	Oil × RES
IW, kg	10.15	10.23	10.2	10.19	0.10			
ADG, kg	0.61	0.59	0.56	0.58	0.01	0.341	0.981	0.450
ADFI, kg	1.21	1.23	1.08	1.08	0.04	0.060	0.851	0.909
F/G	1.98	2.11	1.93	1.87	0.06	0.248	0.791	0.463
FW, kg	26.37	26.74	25.89	26.53	0.45	0.595	0.717	0.889

^1^All of the values are expressed as the means and pooled SEM, *n* = 7, means without a common superscript letter differ (*P* < 0.05)

^2^*IW* Initial weight, *FW* Final weight, *ADG* Average daily gain, *ADFI* Average daily feed intake, *F/G* Average daily feed intake to average daily gain ratio

**Table 2:** Effects of resveratrol (RES) supplementation on nutrient apparent digestibility in oxidized soybean oil (OSO)-challenged weaned piglets^1^

Item^2^	Fresh soybean oil		Oxidized soybean oil		SEM	*P*-value		
	0 mg/kg RES	300 mg/kg RES	0 mg/kg RES	300 mg/kg RES		Oil	RES	Oil × RES
DM, %	91.15	91.21	92.94	91.38	0.32	0.107	0.272	0.233
CP, %	67.63	66.92	68.95	70.44	0.92	0.213	0.838	0.566
EE, %	72.59	77.95	70.48	78.03	1.24	0.657	0.009	0.630
GE, %	77.37	77.90	77.41	77.06	0.53	0.727	0.939	0.702
CF, %	31.51	35.25	32.66	30.98	1.51	0.625	0.746	0.397

^1^All of the values are expressed as the means and pooled SEM, *n* = 7, means without a common superscript letter differ (*P* < 0.05)

^2^*DM* Dry matter, *CP* Crude protein, *EE* Ether extract, *GE* Gross energy, *CF* Crude fiber

**Table 3:** Effects of resveratrol (RES) supplementation on the diarrhea index in oxidized soybean oil (OSO)-challenged weaned piglets^1^

Item	Fresh soybean oil		Oxidized soybean oil		SEM	*P*-value		
	0 mg/kg RES	300 mg/kg RES	0 mg/kg RES	300 mg/kg RES		*P*-Oil	*P*-RES	*P*-diet
D 1 to 7	1.21	1.18	1.26	1.36	0.05	0.989	0.714	0.903
D 8 to 14	1.30	1.52	1.48	1.44	0.10	0.131	0.772	0.185
D 15 to 21	1.35	1.81	1.86	1.198	0.136	0.134	0.639	0.215
D 22 to 28	1.31	1.28	1.24	1.56	0.058	0.772	0.772	0.875

^1^Diferent lowercase letters represent signifcant diference at *P* < 0.05, and *P*-diet represents the *P* values between the four groups. *P*-Oil stands for *P* value between fresh soybean oils groups and oxidized soybean oils groups. *P*-RES stands for *P* value between resveratrol groups and non-resveratrol groups

**Fig. 1:**
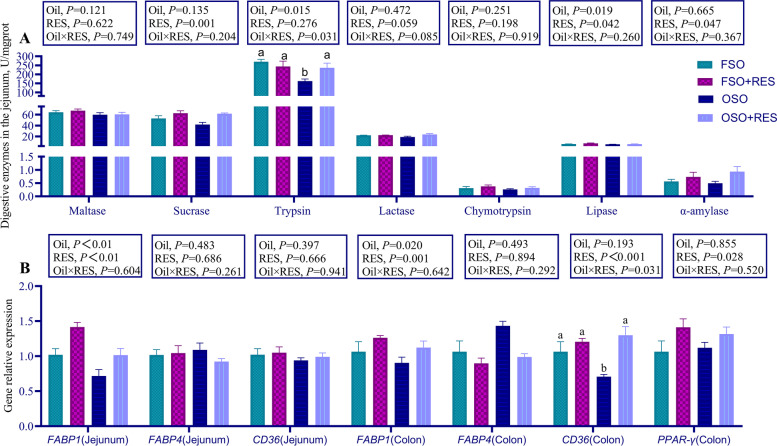
Effect of RES supplementation on activities of the jejunum digestive enzymes and mRNA expression of the jejunum and the colon fat transporter genes in OSO-challenged weaned piglets. **A** Digestive enzymes activity in the jejunum. **B** The mRNA expression of fat transporter genes in the jejunum and colon. The column and its bar represent the mean value and standard error (*n* = 7 piglets/group), respectively; labeled means in a row without a common letter differ, *P* ≤ 0.05; Note: FSO, fresh soybean oils; FSO + RES, fresh soybean oils with resveratrol; OSO, oxidized soybean oil; OSO + RES, oxidized soybean oil with resveratrol

Then, changes in small intestinal digestive enzymatic activity levels were further analyzed (as shown in Fig. 1A). While the OSO supplemented piglets had a lower the activity of lipase compared to FSO diets (*P* < 0.05), Additionally, relative to the non-RES group, dietary RES improved the activity levels of sucrase, lipase, and α-amylase in the jejunum of weaned piglets (*P* < 0.05). Relative to the OSO group, dietary RES supplementation in the diets supplemented with OSO increased the activity levels of trypsin in the jejunum of weaned piglets but failed to influence the activity levels of trypsin when diets were supplemented with FSO (interaction, *P* < 0.05). The mRNA expression of *FABP1* in the jejunum (*P* < 0.05) decreased in weaned piglets fed OSO diets relative to those fed FSO diets. Relative to the non-RES group, dietary RES supplementation increased the *FABP1* mRNA expression levels in the jejunum, the mRNA expression levels of *FABP1*, *CD36*, and *PPAR-γ* in the colon for weaned piglets (*P* < 0.05, Fig. 1B).

### Redox status and immunity in the plasma

**Fig. 2:**
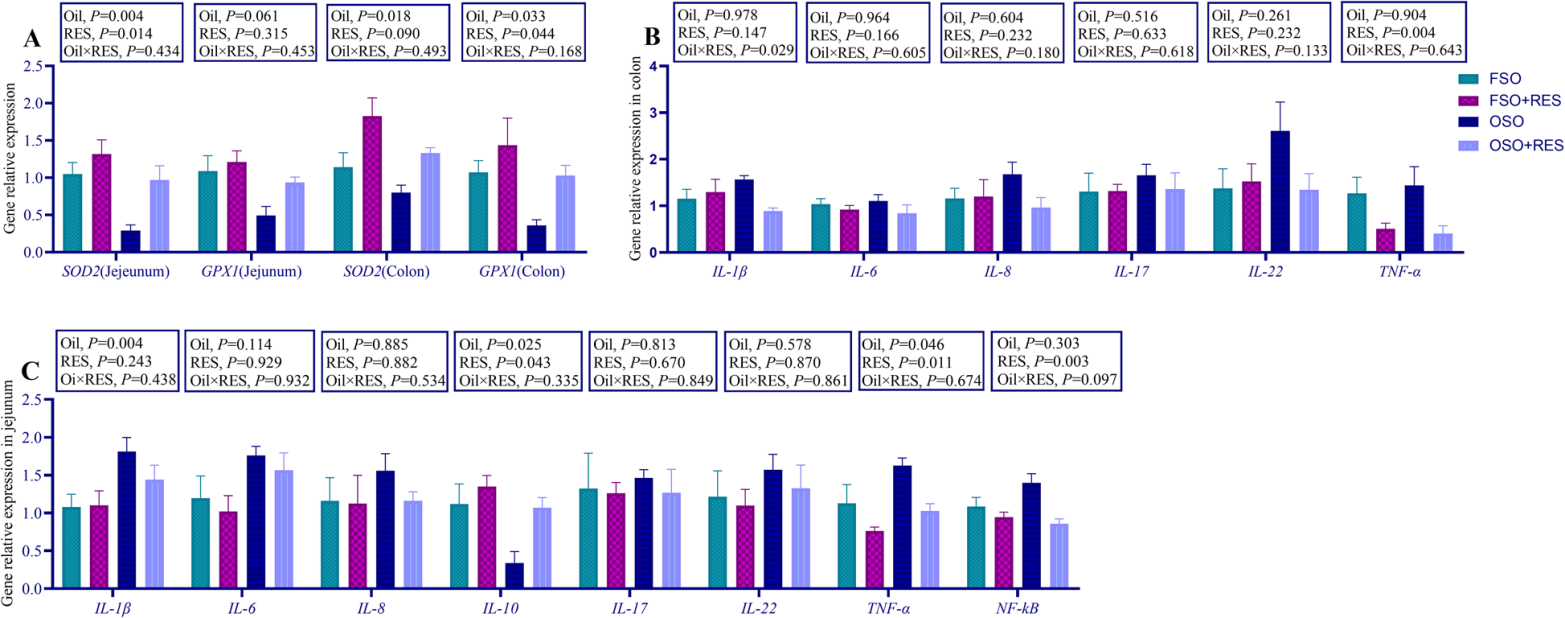
Effect of RES supplementation on the mRNA expression of jejunm, colon oxidative stress genes, inflammatory factors of the jejunum and colon in OSO-challenged weaned piglets. **A** The mRNA expression of oxidative stress genes in the jejunum and colon; **B** The mRNA expression of inflammatory factors of the colon; **C** The mRNA expression of inflammatory factors of the jejunum. The column and its bar represent the mean value and standard error (*n* = 7 piglets/group), respectively; labeled means in a row without a common letter differ, *P* ≤ 0.05; Note: FSO, fresh soybean oils; FSO + RES, fresh soybean oils with resveratrol; OSO, oxidized soybean oil; OSO + RES, oxidized soybean oils with resveratrol

The effects of dietary supplementation on the antioxidant status to d 28 weaned piglets are presented in Table 4. Overall the RES supplemented piglets had increased the activity levels of T-SOD in the plasma compared to the non-RES group during the 28 d experimental period (*P* < 0.05). GSH-Px and T-AOC activity and MDA levels were not influenced by inclusions of OSO or RES (*P* > 0.05). Relative to the OSO group, dietary RES supplementation in the diets supplemented with OSO tended to decrease the levels of H_2_O_2_ in the plasma, the levels of H_2_O_2_ in the plasmas of weaned piglets were increased when diets were supplemented with FSO (interaction, *P* < 0.05). Furthermore, the genes related to antioxidants in the intestine were measured, as shown in Fig. 2A, the mRNA expression of *SOD2* in the jejunum and the mRNA expression of *SOD2* and glutathione peroxidase 1 (*GPX1*) in the colon was decreased by OSO relative to FSO. The mRNA expression of *SOD2* in the jejunum and the mRNA expression of *GPX1* in the colon was increased by RES relative to non-RES (*P* < 0.05). Furthermore, considering that oxidative stress within the organism could induce the production of inflammation, as shown in Table 5, dietary RES supplementation in diets supplemented with FSO increased the levels of IL-6, IL-8, and TNF-α in the plasma of weaned piglets. Relative to the FSO group, supplementation of RES in OSO decreased the levels of IL-1β, IL-6, IL-8, and TNF-α (interaction, *P* < 0.01).

**Table 4:** Effects of resveratrol (RES) supplementation on levels of plasma antioxidant capacity in oxidized soybean oil (OSO)-challenged weaned piglets^1^

Item^2^	Fresh soybean oil		Oxidized soybean oil		SEM	*P*-value		
	0 mg/kg RES	300 mg/kg RES	0 mg/kg RES	300 mg/kg RES		Oil	RES	Oil × RES
H_2_O_2_, mmol/L	64.94^b^	74.93^ab^	82.99^a^	70.11^ab^	2.44	0.138	0.740	0.015
GSH-Px, U/mL	517.07	533.86	554.34	562.69	14.95	0.307	0.694	0.895
T-SOD, U/mL	46.69	68.29	71.61	88.19	5.12	0.019	0.040	0.772
MDA, nmol/mL	3.90	4.22	4.59	4.88	0.20	0.096	0.385	0.863
T-AOC, U/mL	0.36	0.34	0.38	0.37	0.01	0.143	0.978	0.783

^1^All of the values are expressed as the means and pooled SEM, *n* = 7. ^a,b^Means without a common superscript letter differ (*P* < 0.05)

^2^*H*_*2*_*O*_*2*_ Hydrogen peroxide, *GSH-Px* Glutathione peroxidase, *T-SOD* Total superoxide dismutase, *MDA* Malondialdehyde, *T-AOC* Total antioxidant capacity

**Table 5:** Effects of resveratrol (RES) supplementation on levels of plasma inflammatory in oxidized soybean oil (OSO)-challenged weaned piglets ^1^

Items^2^	Fresh soybean oil		Oxidized soybean oil		SEM	*P*-value		
	0 mg/kg RES	300 mg/kg RES	0 mg/kg RES	300 mg/kg RES		Oil	RES	Oil × RES
IL-1β, pg/mL	25.23^b^	28.61^b^	32.43^a^	20.93^c^	1.01	0.841	0.002	< 0.001
IL-6, pg/mL	77.65^c^	121.68^b^	141.78^a^	69.14^d^	6.07	0.036	< 0.001	< 0.001
IL-8, pg/mL	32.71^b^	36.62^ab^	43.78^a^	29.19^b^	1.57	0.040	0.166	< 0.001
TNF-α, pg/mL	46.61^b^	54.75^a^	67.02^a^	39.42^b^	2.68	0.499	0.015	< 0.001
IgA, g/L	1.05	1.01	1.01	1.08	0.04	0.863	0.877	0.557
IgG, g/L	19.17	18.77	17.27	19.73	1.08	0.840	0.655	0.535
IgM, g/L	2.39	2.20	2.17	2.45	0.13	0.943	0.862	0.386

^1^All of the values are expressed as the means and pooled SEM, *n* = 7. ^a,b,c^Means without a common superscript letter differ (*P* < 0.05)

^2^*IL-1β* Interleukin-1β, *TNF-α* Tumor necrosis factor-α, *IL-6* Interleukin-6, *IL-8* Interleukin-8, *IgA* Immunoglobulin A, *IgG* Immunoglobulin G, *IgM* Immunoglobulin M

As shown in Fig. 2, the mRNA expression levels of proinflammatory cytokines, relative to the FSO group, OSO supplementation increased the mRNA expression of *IL-1β, TNF-α,* and decreased the mRNA expression of interleukin-10 (*IL-10*) in the jejunum of weaned piglets (*P* < 0.05). The RES supplemented piglets had increased the mRNA expression of *IL-10* in the jejunum, decreased the mRNA expression of *TNF-α*, nuclear factor κB (*NF-κB*) in the jejunum and *TNF-α* in the colon compared to the non-RES group during the 28 d experimental period (*P* < 0.05). Relative to the FSO group, dietary RES supplementation in the diets supplemented with OSO tended to decrease the mRNA expression levels of *IL-1β* in the colon of weaned piglets but failed to influence the mRNA expression levels of *IL-1β* when diets were supplemented with FSO (interaction, *P* < 0.05, Fig. 2B).

### Intestinal morphology and function

**Fig. 3:**
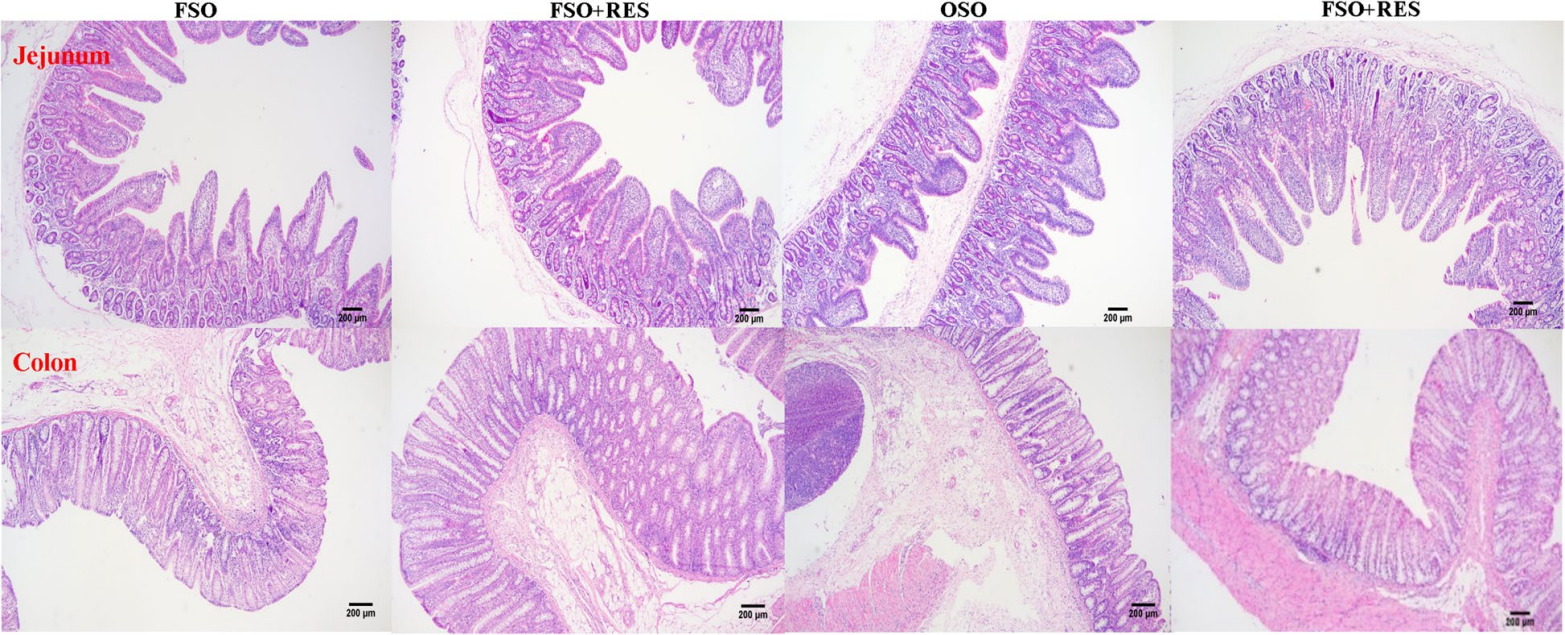
Effect of RES supplementation on intestinal morphology in OSO-challenged weaned piglets FSO, fresh soybean oil; FSO + RES, fresh soybean oils with resveratrol; OSO, oxidized soybean oil; OSO + RES, oxidized soybean oil with resveratrol. Note: Original magnification 200 × , scale bar 200 μm

**Fig. 4:**
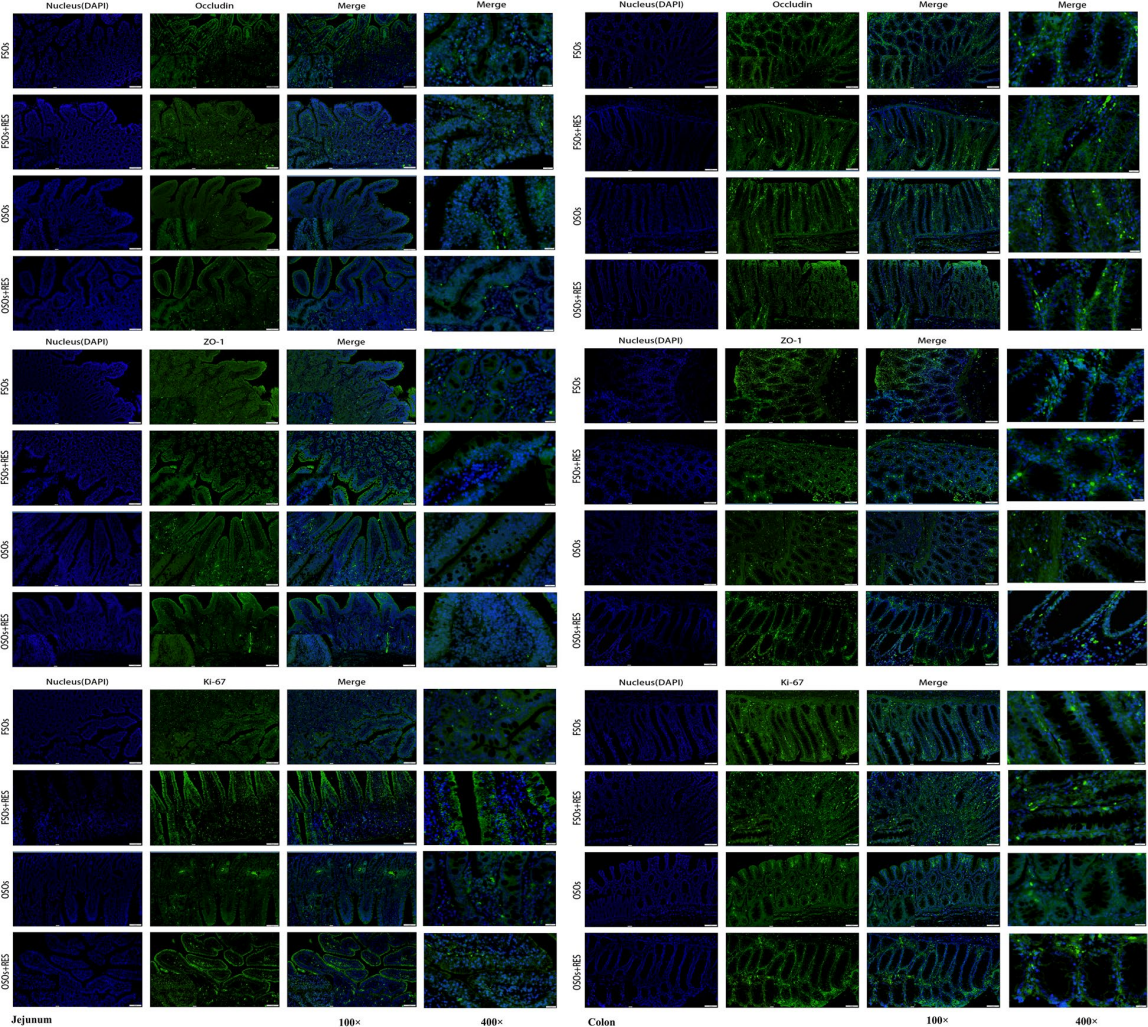
ZO-1, Occludin, Ki-67 staining on immunofluorescence images in paraformaldehyde-fixed cross-sections from the jejunum and the colon of weaned piglets. FSO, fresh soybean oil; FSO + RES, fresh soybean oil with resveratrol; OSO, oxidized soybean oil; OSO + RES, oxidized soybean oils with resveratrol. Note: 100 × and 400 × magnification, scale bar, 200 μm

**Fig. 5:**
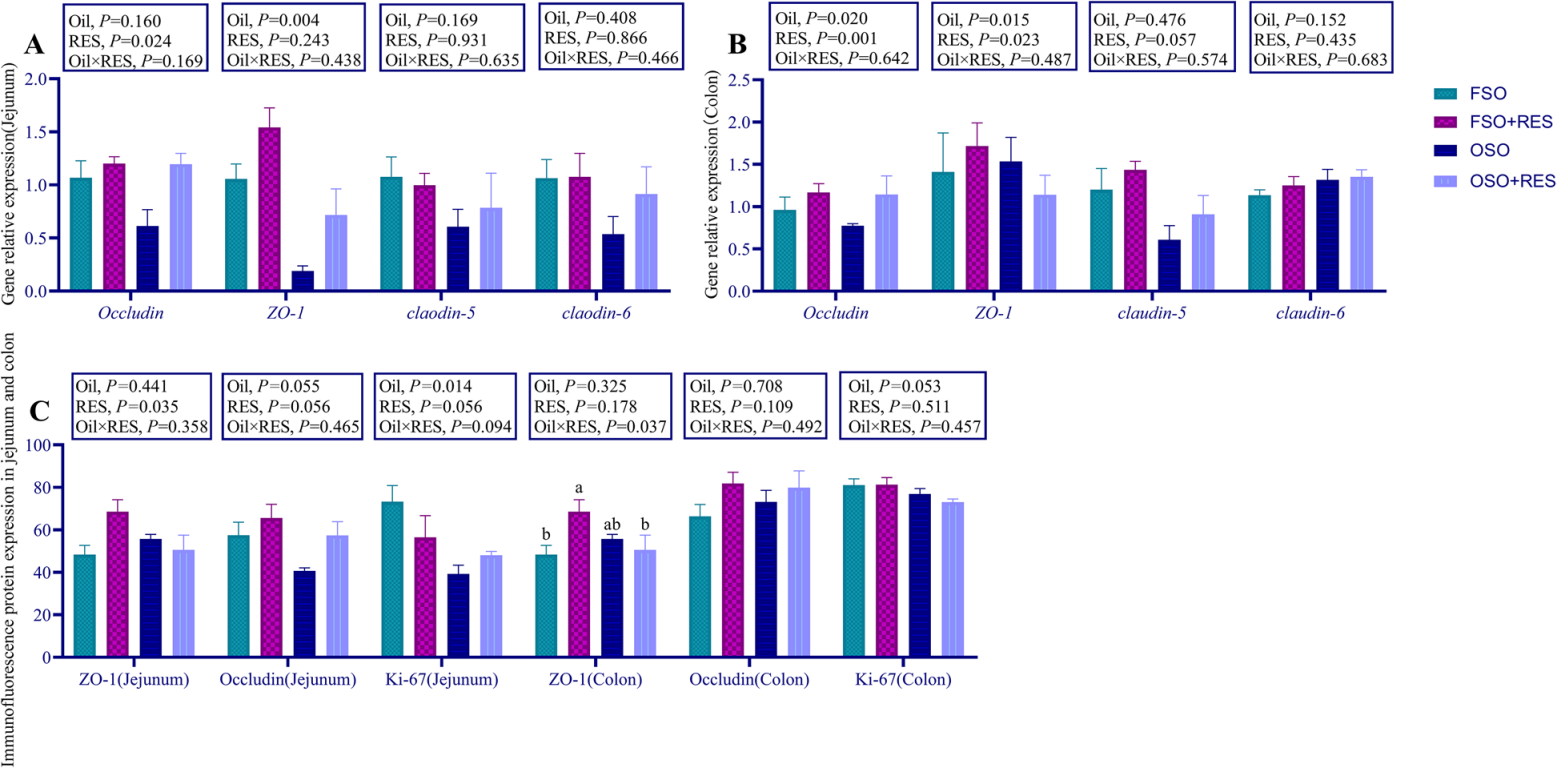
Effect of RES supplementation on immunofluorescence images of the jejunum, colon *ZO-1*, Occludin, Ki-67 and mRNA expression of the jejunum and colon *ZO-1*, *Occludin*, *Claodin-5*, *Claodin-6* genes in OSO-challenged weaned piglets. **A** The mRNA expression of *ZO-1, Occludin*, *Claodin-5*, *Claodin-6* in the jejunum; **B** The mRNA expression of *ZO-1, Occludin, Claodin-5, Claodin-6* in the colon; **C** ZO-1, Occludin, Ki-67 staining on immunofluorescence images in the jejunum and colon; The column and its bar represent the mean value and standard error (*n* = 3 piglets/group), respectively; labeled means in a row without a common letter differ, *P* ≤ 0.05; Note: FSO, fresh soybean oil; FSO + RES, fresh soybean oil with resveratrol; OSO, oxidized soybean oil; OSO + RES, oxidized soybean oil with resveratrol

As shown in Table 6, Figs. 3, 4 and 5, villus/crypt ratios (VCRs) in the jejunum, the mRNA expression levels of *ZO-1* in the jejunum and occludin, *ZO-1* in the colon were decreased by OSO relative to FSO (*P* < 0.05). Relative to the non-RES group, dietary RES increased increased the VH and VCR in the jejunum, and increased the mRNA expression levels of occludin in the jejunum and occludin and *ZO-1* in the colon of weaned piglets (*P* < 0.05). Relative to the OSO group, supplementation with RES in the OSO diet improved the VH in the jejunum, while the VH failed to affect in weaned piglets fed supplementation with RES in the FSO diet relative to those fed FSO diets (interaction, *P* < 0.05). Inclusions of OSO or RES in diets failed to influence the CD in the colon. The mRNA expression levels of Claudin-5 and Claudin-6 in the jejunum and colon were not influenced by inclusions of OSO or RES (*P* > 0.05).

**Table 6:** Effects of resveratrol (RES) supplementation on intestinal morphology in oxidized soybean oil (OSO)-challenged weaned piglets^1^

Items^2^	Fresh soybean oil		Oxidized soybean oil		SEM	*P*-value		
	0 mg/kg RES	300 mg/kg RES	0 mg/kg RES	300 mg/kg RES		Oil	RES	Oil × RES
VH (Jejunum), μm	682.58^ab^	666.73^ab^	571.36^b^	730.22^a^	19.43	0.486	0.044	0.016
CD (Jejunum), μm	492.66	509.63	483.95	551.88	17.24	0.636	0.237	0.474
VCR (Jejunum)	1.40	1.64	1.18	1.35	0.05	0.009	0.032	0.728
CD (Colon), μm	734.03	771.87	715.09	706.85	11.12	0.060	0.493	0.289

^1^All of the values are expressed as the means and pooled SEM, *n* = 3. ^a,b^Means without a common superscript letter differ (*P* < 0.05)

*VH* Villus height, *CD* Crypt depth, *VCR* Jejunum villus/crypt ratios

The immunofluorescence results in the jejunum and colon of weaned piglets are shown in Fig. 5C. The protein expression levels of Ki-67 in the jejunum was decreased by OSO relative to FSO (*P* < 0.05). Relative to the non-RES group, dietary RES increased increased the protein expression levels of ZO-1 in the jejunum of weaned piglets (*P* < 0.05). Relative to the OSO group, supplementing RES in the FSO diet improved the protein expression levels of ZO-1 in the colon of weaned piglets; supplementing RES in the OSO diet had no influence on ZO-1 protein expression relative to those fed FSO diets (interaction, *P* < 0.05).

As shown in Table 7, relative to the non-RES group, dietary RES decreased the levels of *D*-lactic acid in the plasma of weaned piglets (*P* < 0.05). Relative to the FSO group, supplementation of RES in the OSO diet decreased the activity levels of DAO in the plasma, and supplementation of RES in the FSO diet failed to influence it (interaction, *P* < 0.05).

**Table 7:** Effects of resveratrol (RES) supplementation on activities of plasma DAO and levels of *D*-lactate in oxidized soybean oil (OSO)-challenged weaned piglets ^1^

Item^2^	Fresh soybean oil		Oxidized soybean oil		SEM	*P*-value		
	0 mg/kg RES	300 mg/kg RES	0 mg/kg RES	300 mg/kg RES		Oil	RES	Oil × RES
*D*-lactic acid, U/L	3244.47	2334.19	3573.74	3105.71	176.51	0.104	0.045	0.503
DAO, U/L	3.20^b^	3.09^b^	5.29^a^	3.54^b^	0.24	0.001	0.015	0.029

^1^All of the values are expressed as the means and pooled SEM, *n* = 7. ^a,b^Means without a common superscript letter differ (*P* < 0.05)

^2^*DAO* Diamine oxidase

### Microbiota and SCFAs levels in the colonic digesta

**Fig. 6:**
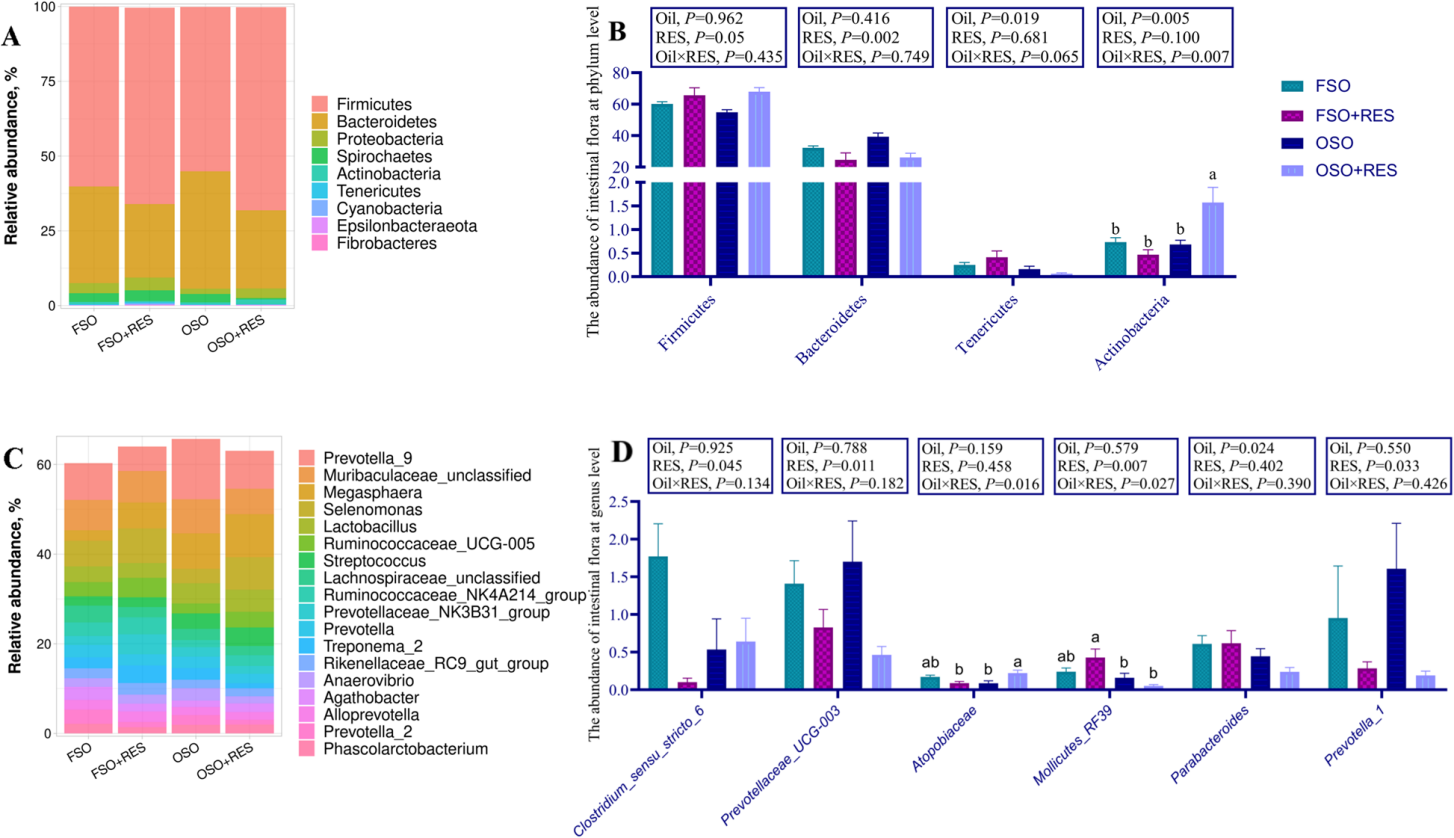
Effect of RES supplementation on the phylum and genus of the colon digesta microbiota in OSO-challenged weaned piglets. **A** At the phylum; **B** The abundance of intestinal flora at phyum level; **C** At the genus; **D** The abundance of intestinal flora at genus level. The column and its bar represent the mean value and standard error (*n* = 7 piglets/group), respectively; labeled means in a row without a common letter differ, *P* ≤ 0.05; Note: FSO, fresh soybean oil; FSO + RES, fresh soybean oil with resveratrol; OSO, oxidized soybean oil; OSO + RES, oxidized soybean oil with resveratrol

As shown in Fig. 6B, at the phylum level, dietary supplementation with OSO decreased the relative abundance of Tenericutes in the colonic digesta compared to the FSO (*P* < 0.05). Relative to the non-RES group, dietary RES increased the abundance of Firmicutes and decreased the abundance of Bacteroidetes of weaned piglets (*P* < 0.05). Relative to the OSO group, dietary supplementation of RES in the OSO diet increased the abundance of Actinobacteria in the colon of weaned piglets, however, RES supplementation in the FSO diet had no influence on Actinobacteria abundance relative to those fed OSO diets (interaction, *P* < 0.05). Furthermore, at the genus level (Fig. 6D), dietary supplementation with OSO decreased the relative abundance of *Parabacteroides* in the colonic digesta compared to the FSO (*P* < 0.05). Relative to the non-RES group, dietary RES decreased the abundance of *Prevotella_1, Clostridium_sensu_stricto_6,* and *Prevotellaceae_UCG003* in the colonic digesta of weaned piglets (*P* < 0.05). Relative to the OSO group, supplementation with RES in the OSO diet tended to increase the *Atopobiaceae* abundance in the colon of weaned piglets; supplementation with RES in the FSO group tended to decrease the *Atopobiaceae* abundance relative to those fed OSO diets(interaction, *P* < 0.05). Relative to the OSO group, supplementation of RES in the FSO diet increased the abundance of *Mollicutes_RF39_unclassified* in the colonic digesta, supplementation of RES in the OSO group failed to influence this abundance (interaction, *P* < 0.05). The alpha-diversity results of weaned piglets were not influenced by inclusions of OSO or RES (Additional file 1: Table S3, *P* > 0.05).

**Fig. 7:**
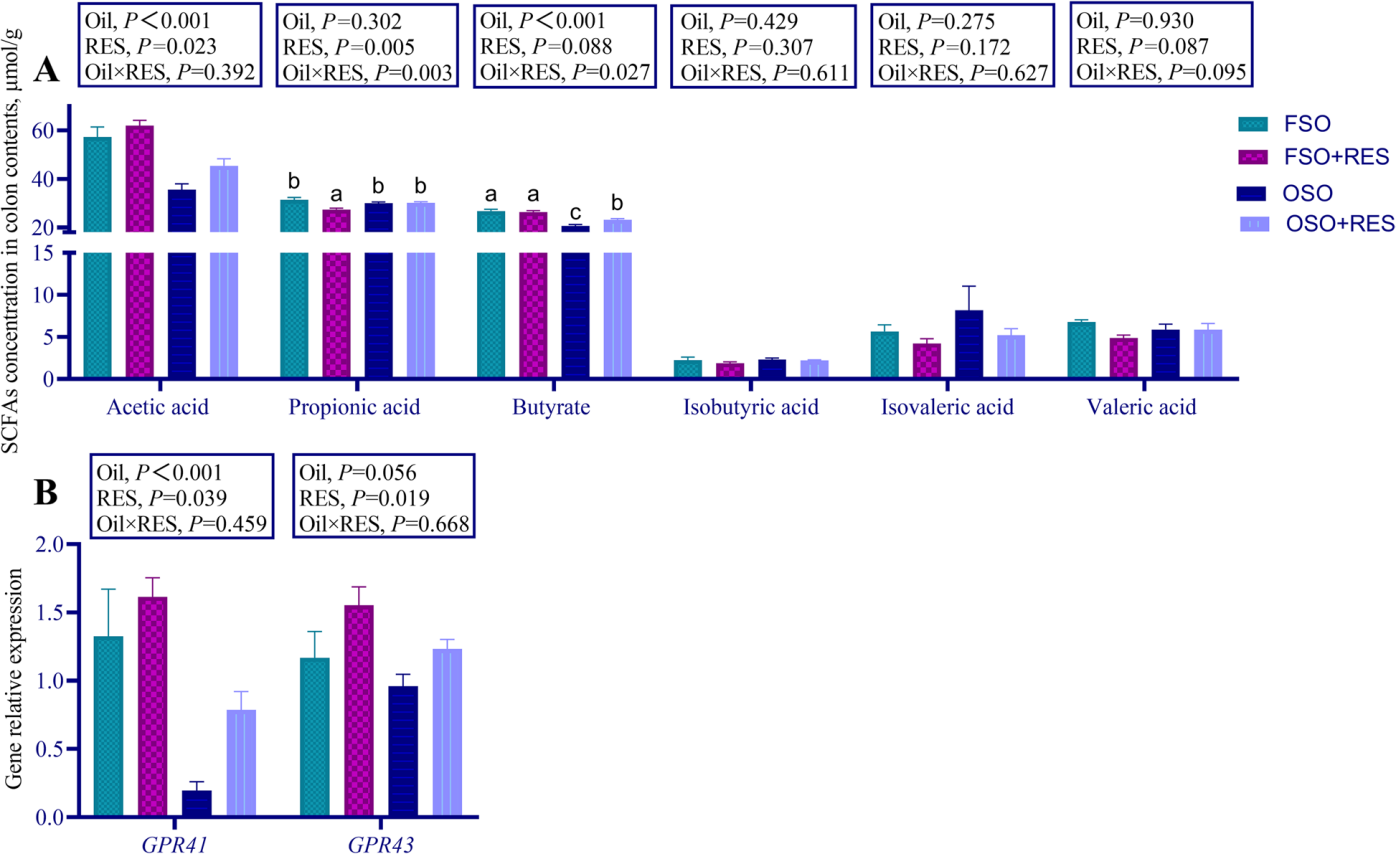
Effect of RES supplementation on levels of the colonic digesta SCFAs and mRNA expression of colon *GPR41*, *GPR43* in OSO-challenged weaned piglets. **A** The levels of SCFAs in colon digesta; **B** The mRNA expression of *GPR41*, *GPR43* in colon. The column and its bar represent the mean value and standard error (*n* = 7 piglets/group), respectively; labeled means in a row without a common letter differ, *P* ≤ 0.05; Note: FSO, fresh soybean oil; FSO + RES, fresh soybean oil with resveratrol; OSO, oxidized soybean oil; OSO + RES, oxidized soybean oil with resveratrol

The levels of SCFAs in the colon of weaned piglets are shown in Fig. 7A. Relative to the FSO group, dietary supplementation with OSO decreased the levels of acetic acid in the colonic digesta compared to the FSO (*P* < 0.05). The levels of acetic acid in the colon of weaned piglets were increased by dietary RES to non-RES (*P* > 0.05). Relative to the FSO group, dietary RES supplementation in the diets supplemented with FSO decreased the level of propionic acid, however, RES supplementation failed to influence the level of propionic acid when the diet was supplemented with OSO (interaction, *P* < 0.01). Relative to the FSO group, dietary RES supplementation in the diets supplemented with OSO increased the levels of butyric acid in the colon of weaned piglets but failed to influence the levels of butyric acid when diets were supplemented with FSO (interaction, *P* < 0.05). The mRNA expression levels of G-protein-coupled receptor-41 (*GPR41*) in the colon of weaned piglets were decreased by OSO relative to FSO (*P* < 0.01), and the mRNA expression levels of *GPR41* and G-protein-coupled receptor-43 (*GPR43*) were increased by dietary RES relative to non-RES (*P* < 0.05, Fig. 7B).

### Correlation analysis of the gut microbiota and variables related to intestinal barrier function, inflammation, oxidative damage and other indicators in weaned piglets

**Fig. 8:**
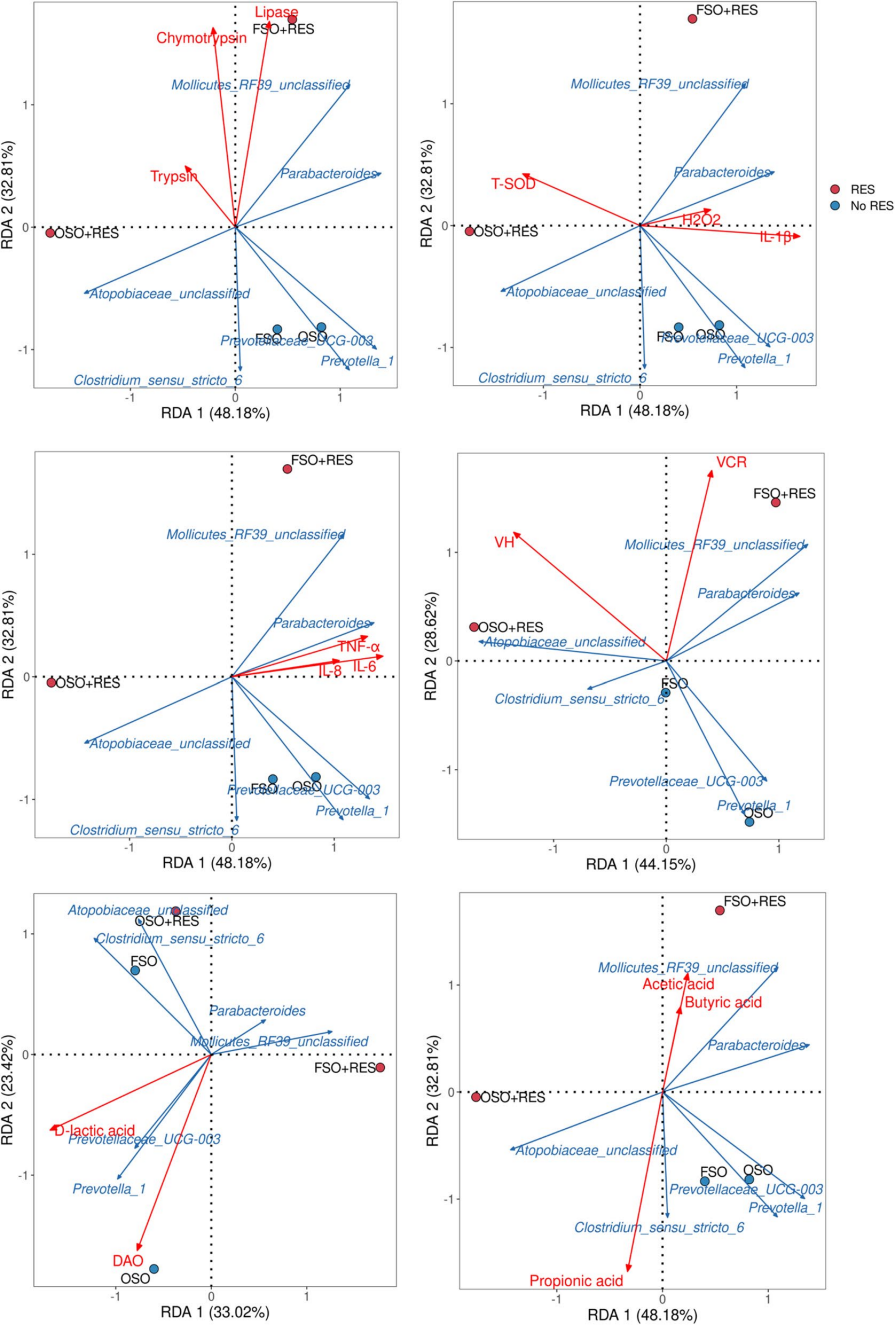
RDA analyses based on the identified differential genera of RES and OSO revealed significantly altered apparent digestibility, proinflammatory factor, and intestinal enzymatic activity-related indices. Note: FSO, fresh soybean oil; FSO + RES, fresh soybean oil with resveratrol; OSO, oxidized soybean oil; OSO + RES, oxidized soybean oil with resveratrol. For the relationship between groups and the identified altered phenotypes, if the plot projection of one sample occurred in the positive direction of the extending line of the identified altered phenotypes, then the treatment of this sample could promote this phenotypic change

RDAs were performed to identify the relationships among the identified differential genera of microbiota, weaned piglets’ performance and treatments (Fig. 8). As a result, we found that the FSO + RES and OSO + RES groups of weaned piglets were all separately clustered in the area near the improved digestibility of short-chain fatty acid content (*P* < 0.05), while the FSO and OSO groups were not well clustered, which indicated that supplementation with RES was beneficial to microbiota fermentation, which was related to the increased the contents acetic, butyric, propionic acid of weaned piglets. However, the OSO groups of weaned piglets were all separately clustered in the area near intensified inflammatory cytokines, the contents of *D*-lactic acid in plasma (*P* < 0.05), while the FSO + RES and OSO + RES groups were not well clustered. These results indicated that supplementation with OSO resulted in decreased the abundance of *Parabacteroides*, which intensified the inflammatory state. Furthermore, according to the Pearson correlation analyses, several key genera that were separately correlated with the digestibility of EE, lipase, trypsin, α-amylase, and chymotrypsin; the VH and VCR in the jejunum; T-SOD, H_2_O_2_, IL-1β, IL-6, IL-8, TNF-α, *D*-lactic acid, and DAO in the plasma; and propionic acid and butyric acid in the colonic digesta were also further identified and are listed in Table 8. Several different genera in the four treatment groups may affect the intestinal digestive enzyme activities, apparent digestibility and proinflammatory factors. Specifically, of these genera, *Prevotellaceae_UCG-003* and *Prevotella_1* were negatively correlated with the increase in EE in weaned piglets (*P* < 0.05). The abundance of *Prevotellaceae_UCG_003* was negatively associated with the changes in α-amylase (*P* < 0.05). The abundance of *Atopobiaceae_unclassified* was negatively correlated with the increase in IL-6 in the plasma of weaned piglets (*P* < 0.05).

**Table 8:** Pearson correlation analyses between key genera and growth performance, nutrient apparent digestibility, intestinal histomorphology, antioxygenic properties, inflammatory cytokines, intestinal enzymatic activities and SCAFs

Genus	Performance	Correlation coefficient	*P*-value
*Prevotellaceae_UCG-003*	α-amylase	−0.990	0.010
*Prevotella_1*	EE	−0.981	0.019
*Prevotellaceae_UCG-003*	EE	−0.966	0.034
*Atopobiaceae_unclassified*	IL-6	−0.954	0.046
*Atopobiaceae_unclassified*	IL-1β	−0.947	0.053
*Prevotellaceae_UCG-003*	CD (Jejunum)	−0.941	0.059
*Prevotella_1*	α- amylase	−0.914	0.086
*Atopobiaceae_unclassified*	TNF-α	−0.909	0.091
*Mollicutes_RF39_unclassified*	CD (Colon)	0.902	0.098
*Atopobiaceae_unclassified*	IL-8	−0.887	0.113
*Clostridium_sensu_stricto_6*	Propionic acid	0.869	0.131
*Prevotella_1*	VH	−0.864	0.136
*Mollicutes_RF39_unclassified*	Lipase	0.861	0.139
*Parabacteroides*	T-SOD	−0.844	0.156

## Discussion

Kerr et al. [41] found that the change in fatty acid composition and/or the presence of lipid peroxidation products in peroxidized oxidized soybean oil may reduce ADG and ADFI values in nursery pigs (initial body weight 7.1 ± 0.9 kg). Some studies have reported that dietary oxidized fish oil decreases average daily gain, significantly increases F/G, decreases the apparent digestibility levels of nutrients from dry matter and ether extract, and increases the diarrhea indices of weaned piglets [42]. However, no effects of oxidized soybean oil on the growth performance levels of piglets are found in our study, similar to the results of Gao et al. [43], who found that dietary supplementation with oxidized soybean oil does not affect the performance levels of sows [43]. This suggests that the weaned piglet body itself may have a certain regulatory role to cope with the stress caused by low oxidation degree of oil. Interestingly, in this study, it is found that dietary oxidized soybean oil tends to decrease the ADFI of weaned piglets. We speculate that there are two reasons for this phenomenon. First, the presence of lipid peroxides affects the palatability of diets, which may occur because lipid peroxides, polar and nonpolar acids, ketones and aldehydes are generated during the oxidation of oils. These substances can produce various odors and reduce the palatability of diets. Second, lipid peroxidation products have somewhat damaged the gastrointestinal tracts of animals, changing the function of the cell membrane, thus damaging the digestive and absorption functions [44]. Zeng et al. [45] reported that dietary RES has no significant effect on the growth performance levels of weaned piglets, but dietary RES increases the expression levels of slow MyHC, the activity levels of succinic dehydrogenase and malate dehydrogenase and the proportion of type I fiber; additionally, it decreases the activity levels of lactate dehydrogenase and the proportion of type II fiber, improving meat quality. RES is known to have many properties, including bactericidal and anti-inflammatory properties. Thus, it can prevent diarrhea in piglets. However, no effects of resveratrol on the growth performance and diarrhea index values of piglets are found in our study, which is consistent with Zhang et al. [46]. This phenomenon suggests that the effects of RES on the growth performance levels of animals may vary with individual differences, feeding methods, dosages and stressors.

In this experiment, the apparent digestibility DM, CP, GE, and CF of finishing pigs was not significantly affected by dietary oxidized oil. Koo et al. [47] found that the influence of oxidized oil on microbial activity was greater than that on nutrient digestibility, which also indicated that the oxidized oil did not necessarily have harmful effects on animal performance, and the growth rate of livestock and poultry might be reduced only when the oxidized racidity threshold was exceeded. Gan et al. [48] found that the apparent digestibility of fatty nutrients of piglets fed 300 mg/kg RES was higher than that of the control group. Our results are similar to those of Kanazawa et al. [49], who reported that lipid peroxidation impaired the functionality of the intestine, this phenomenon is indicated by decreased enzyme activity (sucrase, maltase and alkaline phosphatase) in the jejunum and ileum after the oral administration of products of linoleic acid peroxidation to rats. Ki-67 is a specific and reliable marker of cell proliferation [50]. Wang et al. [51] indicated that the proliferation of epithelial cells may have a direct impact on the digestive and absorptive functions of the small intestine; the researchers have found that the number of Ki-67-positive cells is related to lactase activity. Therefore, our findings indicate that OSO may impair the digestive capacity of the GI tract by inhibiting the proliferation of intestinal epithelial cells.

In this study, we find that RES increases the mRNA expression levels of *GPR41* and *GPR43* in piglets, which are the only two short-chain fatty acid-specific receptors that have been discovered [52]. These receptors can inhibit the activity of histone deacetylase by mediating short-chain fatty acid signaling [53], and they play important roles in the absorption of nutrients in animal intestines. Studies have shown that genetically modified mice deficient in GPR43 are obese on a normal diet, whereas mice that overexpress GPR43 specifically in adipose tissue are protected against diet-induced obesity [54, 55]. In addition, a clinical study shows that propionic and butyric acid promote the formation of porcine adipocytes and increase the expression levels of PPAR-γ, which is a key transcription factor controlling lipid homeostasis; it also increases the mRNA expression level of *CEBPA* in the interstitial vascular region [56]. FABP1 plays a significant role in the normal lipid metabolism of differentiated intestinal epithelial cells, especially regarding the uptake and basolateral secretion of fatty acids [56]. Furthermore, the CD36 receptor, as a multifunctional membrane receptor, can effectively promote fatty acid uptake and improve lipid metabolism [57]. Our previous studies have shown that maternal dietary RES increases the fat contents in the *longissimus pectoris* and in milk [23, 58]. It has been reported that adding RES to feed increases the fatty acid oxidation and energy release rates [59].

Lipid peroxidation products ingested by animals often disrupt the redox balance and induce inflammation, resulting in a lack of antioxidant systems in the intestine and other tissues [5, 7]. Similarly, a study by Yan et al. [13] showed that dietary supplementation with 5% oxidized soybean oil reduces the activity levels of SOD and the contents of GPx and increases the contents of MDA in the jejunum of piglets. It has been reported that dietary oxidized fish oil decreases the serum T-AOC contents and T-SOD activity levels and increases the serum MDA contents in in serum of weaned piglets [42]. Zhang  et al. [17] reported that adding RES to diets improves the activity levels of T-AOC and GSH-Px and the mRNA expression content in the *longissimus dorsi* muscles of finishing pigs, improving pork quality. Consistent with these findings, the results in the present study show that dietary RES further increases T-SOD activity levels in the plasmas of weaned piglets in the OSO-challenged group. As a central redox signaling molecule, H_2_O_2_ induces oxidative stress in the gut epithelium [60]. Dietary RES with OSO reduces the levels of H_2_O_2_ in the plasmas of weaned piglets. Therefore, dietary RES partially prevents OSO-challenged diet-induced oxidative stress and metabolic dysfunction. As expected, aside from its antioxidant effect, RES promotes increases in antioxidant genes in the gut of OSO-challenged piglets.

Intestinal morphology is an important indicator of intestinal health, and it is usually evaluated according to VH, CD, VCR and other indicators [61]. Previous study have reported that RES repairs the damaged villus-crypt structure and benefits intestinal absorption characteristics in piglets [18]. As expected, our results showed that RES supplementation increased VH and VCR values in the jejunum, indicating that RES was beneficial for intestinal morphological recovery. Additionally, we have observed that OSO damages the intestinal structures of weaned piglets. Consistent with these findings, a slight decrease in feed intake is observed in weaned piglets fed a diet containing OSO. Previous study have found that low feed intake can reduce VCR values [62]. However, this phenomenon could be related to enterocyte dysfunction and oxidative stress, among other reasons. A previous study found that dietary supplementation with peroxidized dietary lipids causes longer and thinner villi and deeper crypts in the jejunum [25]. Moreover, there is evidence that the induction of oxidative stress and disruption of redox balance in intestinal cells contribute to losses in intestinal integrity and activate proinflammatory transcription factors [63]. Therefore, whether the effects of dietary OSO on intestinal morphology are caused by reduced feed intake or oxidative stress needs to be further studied.

Fukudome et al. [64] indicated that the level of plasma DAO activity is associated with the maturation and integrity of small intestinal mucosa. It has been reported that increase in *D*-lactate may reflect an efflux of bacteria and/or its products into circulation because of intestinal mucosal injury [65]. When intestinal barrier function is impaired, the intestinal permeability chemical markers *D*-lactic acid and DAO are released into the blood. Consistent with this observation [66], our results show that supplementation with RES decreases the levels of plasma DAO and the activity levels of *D*-lactate. Moreover, RES significantly suppresses the release of plasma DAO in OSO-challenged piglets. One potential explanation for this finding might be the RES-mediated upregulation characteristics of occludin, ZO-1 mRNA and protein in the jejunum of OSO-challenged piglets. Occludin and ZO-1 participate in tight junction assembly and stability, and they are the proteins with the best barrier function [67]. Our results are similar to those of Qiu et al. [66]. Similarly, in previous study, RES treatment increased the epithelial expression and phosphorylation of occludin and ZO-1 in a concentration-dependent manner. Moreover, RES which protected Caco-2 cells from H_2_O_2_-induced oxidative damage clearly reduced malondialdehyde level and intracellular reactive oxygen species accumulation, but increased the expression levels of superoxide dismutase and heme oxygenase-1 [68].

Previous studies have shown that RES is a dietary polyphenol with a variety of intestinal bioactivity levels that has been proven to improve intestinal functions and intestinal microflora distributions in animals [20, 22]. In the present study, an increased relative abundance of Firmicutes and decreased abundance of Bacteroidetes are observed in RES-supplemented piglets. Qiu et al. [66] demonstrated that dietary RES decreases the abundance of Bacteroidetes. There is a correlation between body weight and intestinal microbial ecology. Obese animals have an increased proportion of Firmicutes and a decreased proportion of Bacteroidetes in their gut [69, 70]. Zhang et al. [71] reported that dietary supplementation with 300 and 600 mg/kg RES may significantly reduce the average backfat thicknesses of growing-finishing pigs, but no significant effects are found on pig body weight and carcass weight. These results indicate that RES might have an anti-obesity effect according to the regulation of the ratio of Bacteroidetes to Firmicutes. Supplementation of RES in OSO diets increases the abundance of *Actinobacteria*, which are part of the normal intestinal flora and are rarely observed in the intestinal flora of different gastrointestinal diseases [69]. At the genus level, *Prevotella*, as one of the most predominant genera among the intestinal microbiot in both preweaned and postweaned pigs, may contribute to microbiota-induced mucosal immune development. *Prevotella* spp. may contribute to the maturation of mucosal immunity in several manners [72]. In our study, it is found that dietary RES reduces the abundance levels of *Prevotella-1* and *Prevotellaceae UCG003*. Interestingly, there is growing evidence that *Prevotella* is involved in inflammatory bowel conditions [73]. Notably, these effects may be species- or strain-specific rather than common across all *Prevotella* spp. *Atopobiaceae* inhibits proinflammatory metabolites and reduces systemic inflammation [74]. Supplementation of RES in diets containing OSO increases the abundance levels of *Atopobiaceae_unclassified* in the colon of weaned piglets. In addition, the abundance of *Atopobiaceae_unclassified* is negatively correlated with the increase in IL-6 in the plasma. Previous study have shown that dietary RES supplementation increases intestinal butyric acid and isobutyric acid contents in mice [75]. Butyric acid alleviates intestinal inflammation by decreasing IL-6 and IL-1β and increasing IL-10 levels [76]. In this study, it is found that supplementation with RES in OSO diet could increase the contents of colon butyric acid and the mRNA expression of *IL-10* in the jejunum. Dietary RES supplementation can increase the colonic acetic acid content, which is known to have powerful anti-inflammatory effects [77]. A previous study has reported that RES supplementation prevents TNF-α production in piglets infected with rotavirus [78]. Consistent with this observation, the results in the present study show that dietary RES supplementation reverses the OSO-induced increases in IL-1β, IL-6, IL-8 and TNF-α in the plasmas of weaned piglets, suggesting that providing OSO-challenged piglets with diets containing RES can efficiently inhibit inflammation. In addition, we find that the dietary supplementation of RES with OSO fails to influence the abundance levels of *Mollicutes_RF39_unclassified*, which only increases when diets are supplemented with FSO. According to reports, *Mollicutes_RF39_unclassified* is associated with prostate cancer [79]. Conflicting results may be dose-dependent, with RES showing proliferative activity at low doses (5 mol/L) and pro-apoptotic activity at high doses [15 mol/L (more higher)] in another study of androgen-sensitive prostate cancer cells [80]. Most of its anticancer properties are attributed to its ability to induce apoptosis in cancer cells [81]. Dietary RES reduces the colonic abundance levels of *Clostridium_sensu_stricto_6* bacteria, which is a common cause of diarrhea, in weaned piglets [82]. Dietary supplementation with OSO decreases the abundance of *Parabacteroides* in the colon, which is a genus of Bacteroides, and reduces metabolic dysfunction in mice fed a high-fat diet by converting bile acids and producing succinic acid [83]. In the present study, supplementation with RES reverses the increased levels of IL-1β, IL-6, IL-8 and TNF-α in the plasma that are induced by OSO-challenged diets, which may be supported by the increased abundance of *Atopobiaceae* and the decreased abundance levels of *Prevotella-1* and *Prevotellaceae UCG003.* Altogether, based on these results, we conclude that the RES-mediated alleviation of the OSO-induced inflammatory response and oxidative damage may be linked to changes in the gut microbiota and butyric and acetic acid levels. Differences between individuals in RES absorption, metabolism and excretion determine the pharmacokinetic characteristics of RES. However, further study is warranted to explore the contribution of the gut microbiome to OSO-challenged intestinal inflammation and oxidative damage. In addition, we speculate that RES may increase the mRNA expression of *CD36* and *PPARγ* in the colon, increase the mRNA expression of *FABP1* in the jejunum and increase the butyric acid content, thus alleviating the effects of OSO on the energy metabolism characteristics of weaned piglets.

## Conclusions

The oxidative stress, the local immune system of the intestine, intestinal morphology were impaired by alterations in the colonic microbiota when weaned piglets were fed OSO. The conclusions prompt further study of exactly how partially OSO compromised the intestinal health. Conversely, dietary RES supplementation may play a beneficial role in the intestinal health of piglets affected by OSO-challenged via regulation of the composition of the intestinal microbiota and its metabolite acetic and propionic acid. Future research should further explore the underlying mechanisms that drive the interaction between colon microbiota and metabolites.

## Supplementary Information

**Additional file 1: Table S1.** Composition and the nutrient levels of the diets (%, as-fed basis unless otherwise stated). **Table S2.** Antibody dilution ratio. **Table S3.** Composition of qRT-PCR system. **Table S4.** Program settings of qRT-PCR. **Table S5.** Primers used for real-time PCR. **Table S6.** Effects of RES supplementation on Alpha diversity of fecal microbiota in OSO challenged weaned piglets. **Table S7.** Pearson correlation analyses between key genera and growth performance, nutrient apparent digestibility, intestinal histomorphology, antioxygenic properties, inflammatory cytokines, intestinal enzymatic activities and SCAFs.

## Data Availability

The datasets produced and/or analyzed during the current study are available from the corresponding author on reasonable request.

## Abbreviations

ADG: Average daily gain
ADFI: Average daily feed intake
CP: Crude protein
CD: Crypt depth
CD36: Cluster of differentiation 36
DM: Dry matter
DAO: Diamine oxidase
EE: Crude fat
FSO: Fresh soybean oil
F/G: Average daily feed intake/average daily gain
FABP1: Fatty acid-binding protein-1
FABP4: Fatty acid-binding protein-4
GE: Gross energy
GPR41: G-protein-coupled receptor-41
GPR43: G-protein-coupled receptor-43
GSH-Px: Glutathione peroxidase
H_2_O_2_: Hydrogen peroxide
IL-1β: Interleukin 1β
IL-6: Interleukin 6
IL-8: Interleukin 8
IL-10: Interleukin 10
IL-17: Interleukin 17
IL-22: Interleukin 22
MDA: Malondialdehyde
NF-κB: Nuclear factor κB
OSO: Oxidized soybean oil
PPARγ: Peroxisome proliferator-activated receptor-γ
PUFAs: Polyunsaturated fatty acids
RES: Resveratrol
T-SOD: Total superoxide dismutase
T-AOC: Total antioxidant capacity
TNF-α: Tumor necrosis factor α
VH: Villus height
VCR: Villus/crypt ratio
SCFAs: Short-chain fatty acids
ZO-1: Zonula occludens-1
