# Supplementary material for: Resveratrol alleviates oxidative stress induced by oxidized soybean oil and improves gut function via changing gut microbiota in weaned piglets
Source: J Anim Sci Biotechnol. 2023 Apr 7;14:54. doi: 10.1186/s40104-023-00851-2 (PMC10080898; doi:10.1186/s40104-023-00851-2)
Supplement: Supplementary file 1 — Additional file 1: Table S1. Composition and the nutrient levels of the diets (%, as-fed basis unless otherwise stated). Table S2. Antibody dilution ratio. Table S3. Composition of qRT-PCR system. Table S4. Program settings of qRT-PCR. Table S5. Primers used for real-time PCR. Table S6. Effects of RES supplementation on Alpha diversity of fecal microbiota in OSO challenged weaned piglets. Table S7. Pearson correlation analyses between key genera and growth performance, nutrient apparent digestibility, intestinal histomorphology, antioxygenic properties, inflammatory cytokines, intestinal enzymatic activities and SCAFs. [file 40104_2023_851_MOESM1_ESM.docx]

**Additional file 1: Table S1** Composition and the nutrient levels of the diets (%, as-fed basis unless otherwise stated)

| **Ingredients** | **Fresh soybean oil** | | **Fresh soybean oil** | |
| --- | --- | --- | --- | --- |
|  | **0 mg/kg**  **RES** | **300 mg/kg**  **RES** | **0 mg/kg**  **RES** | **300 mg/kg**  **RES** |
| Corn grain | 63.26 | 63.26 | 63.26 | 63.26 |
| Full-fat soybean | 5.5 | 5.5 | 5.5 | 5.5 |
| Soybean meal | 15.8 | 15.8 | 15.8 | 15.8 |
| Corn gluten meal | 1.4 | 1.4 | 1.4 | 1.4 |
| Whey powder | 4 | 4 | 4 | 4 |
| Fish meal | 3 | 3 | 3 | 3 |
| Fresh soybean oil | 3 | 3 |  |  |
| Oxidized oil |  |  | 3 | 3 |
| Lysine (98%) | 0.65 | 0.65 | 0.65 | 0.65 |
| Methionine (98%) | 0.1 | 0.1 | 0.1 | 0.1 |
| Threonine (98%) | 0.18 | 0.18 | 0.18 | 0.18 |
| Tryptophan (98%) | 0.03 | 0.03 | 0.03 | 0.03 |
| Dicalcium phosphate | 0.9 | 0.9 | 0.9 | 0.9 |
| Limestone | 0.78 | 0.78 | 0.78 | 0.78 |
| NaCl | 0.4 | 0.4 | 0.4 | 0.4 |
| Premix^1^ (1%) | 1 | 1 | 1 | 1 |
| Total |  |  |  | 100.00 |
| Nutrient level^2^ |  |  |  |  |
| Net energy, MJ/kg | 2.59 | 2.59 | 2.59 | 2.59 |
| Crude protein | 18.83 | 18.88 | 19.06 | 19.20 |
| Lysine | 1.44 | 1.44 | 1.44 | 1.44 |
| Tryptophan | 0.23 | 0.23 | 0.23 | 0.23 |
| Threonine | 0.87 | 0.87 | 0.87 | 0.87 |
| Methionine | 0.42 | 0.42 | 0.42 | 0.42 |
| Calcium | 0.7 | 0.7 | 0.7 | 0.7 |
| Total phosphorus | 0.56 | 0.56 | 0.56 | 0.56 |
| **Fatty acid composition, % of total fatty acids^3^** | **Fresh soybean oil** | | **Oxidized soybean oil** | |
| Myristic acid C14:0 | 0.05 | | 0.08 | |
| Palmitic acid C16:0 | 9.61 | | 10.95 | |
| Palmitoleic acid C16:1 | 0.05 | | 0.08 | |
| Margaric acid C17:0 | 0.08 | | 0.09 | |
| Stearic acid C18:0 | 3.66 | | 3.69 | |
| Elaidic acid C18:1T | 18.21 | | 20.10 | |
| Linoleic acid C18:2 | 0.05 | | 0.05 | |
| Linoelaidic acid C18:2TT | 48.44 | | 44.69 | |
| Arachidic acid C20:0 | 0.18 | | 0.16 | |
| Eicosenic *cis* C20:1 | 7.12 | | 6.20 | |
| Arachidonic acid C20:2 | 0.03 | | 0.03 | |
| Behenic acid C22:0 | 0.36 | | 0.34 | |
| Eicosapentaenoic acid C20:5 | 0.11 | | 0.12 | |
| Docosahexaenoic acid C22:6NS | 0.03 | | 0.02 | |
| Total SFA^4^ | 13.95 | | 15.32 | |
| Total MUFA^5^ | 25.38 | | 26.37 | |
| Total PUFA^6^ | 48.86 | | 45.14 | |

^1^ Premix (1%) for per kilogram of diet: VA 22,500 IU, VD_3_ 4,250 IU, VE 40 mg, VK_3_ 2.5 mg, VB_1_ 4 mg, VB_2_ 10 mg, VB_6_ 4 mg, VB_12_ 0.05 mg, Niacin 50 mg, *D*-pantothenic acid 22.5 mg, Folic acid 2 mg, biotin 0.25 mg, Fe (as FeSO_4_·H_2_O) 190 mg, Cu (as CuSO_4_·5H_2_O) 190 mg, Mn (as MnSO_4_·H_2_O) 45 mg, Zn (as ZnSO_4_·H_2_O) 140 mg, Se (as Na_2_SeO_3_) 0.4 mg, I (as Ca(IO_3_)_2_) 0.5 mg

^2^ Crude protein, crude fat, calcium, and total phosphorus were analyzed values, the rest were calculated values

^3^ SFA = saturated fatty acid; MUFA = monounsaturated fatty acid; PUFA = polyunsaturated fatty acid

^4^ Total SFA = [C8:0] + [C10:0] + [C12:0] + [C14:0] + [C16:0] + [C17:0] + [C18:0] + [C20:0] + [C22:0] + [C24:0]

^5^ Total MUFA = [C14:1] + [C16:1] + [C18:1n-9] + [C18:1n-7] + [C20:1] + [C24:1]

^6^ Total PUFA = [C18:2n-6] + [C18:3n-3] + [C18:3n-6] + [C20:2] + [C20:4n-6]

### Additional file 1: Table S2 Antibody dilution ratio

| **Antibody name** | **Dilution ratio** |
| --- | --- |
| *ZO-1* Rabbit pAb | 1:400 |
| *Occludin* Rabbit pAb | 1:400 |
| Mouse Anti-*Ki-67* Monoclonal Antibody | 1:200 |
| DAPI | 1:2,000 |
| Goat Anti-Rabbit *IgG* (H+L) Highly Cross-Adsorbed Secondary Antibody | 1:200 |
| Goat Anti-Mouse *IgG* (H+L) Highly Cross-Adsorbed Secondary Antibody | 1:200 |

**Additional file 1: Table S3** Composition of qRT-PCR system

| **Reaction system composition** | **Dosage** | **Final concentration** |
| --- | --- | --- |
| SYBR® Premix Ex TaqTM (2×) | 10 µL | 1× |
| PCR Forward Primer (10 μmol/L) | 0.4 µL | 0.2 μmol/L |
| PCR Reverse Primer (10 μmol/L) | 0.4 µL | 0.2 μmol/L |
| ROX Reference Dye II (50×) | 0.4 µL | 1× |
| d H_2_O (Sterilize distilled water) | 6.8 µL |  |
| cDNA template120 ng/µL | 2 µL | 12 ng/µL |
| Total | 20 µL |  |

**Additional file 1: Table S4** Program settings of qRT-PCR

| **Item** | **Predegeneration (Stage 1)** | **PCR reaction (Stage 2)** |
| --- | --- | --- |
| Cycles (Reps) | 1 | 40 |
| Melting temperature | 95 °C 10 s | 95 °C 5 s |
| Elongating temperature |  | 60 °C 34 s |

**Additional file 1: Table S5** Primers used for real-time PCR

| **Genes** | **Sequences (5' to 3')** | **Product sizes, bp** | **Gen Bank No.** |
| --- | --- | --- | --- |
| *β-actin* | F: GGCACCACACCTTCTACAACGAG | 102 | XM_003124280.5 |
|  | R: TCATCTTCTCACGGTTGGCTTTGG |  |  |
| *GAPDH* | F: ACTCACTCTTCCACTTTTGATGCT | 150 | NM_008084.3 |
|  | R: TGTTGCTGTAGCCAAATTCA |  |  |
| *FABP1* | F: GGACATCAAGGGGACATCGGAAATC | 121 | NM_001004046 |
|  | R: GGTCTCCATCTCACACTCCTCTCC |  |  |
| *FABP4* | F: TGCAGAAGTGGGATGGAAAGACAAC | 108 | NM_001002817.1 |
|  | R: TCTCATAAATTCTGGTAGCCGTGACAC |  |  |
| *CD36* | F: CGTACAGAGTTCGTTATCTAGCCAAGG | 149 | NM_001044622 |
|  | R: AGCCAGATTGAGAACAGTGAAGGTG |  |  |
| *PPARγ* | F: TCTGTGGACCTGTCGGTGATGG | 122 | NM_214379.1 |
|  | R: TCAGCTCTCGGGAATGGGATGTC |  |  |
| *SOD2* | F: TTTCTGGACAAATCTGAGCCCTAACG | 122 | NM_214127 |
|  | R: CGACGGATACAGCGGTCAACTTC |  |  |
| *GPX1* | F: GGAGATCCTGAATTGCCTCAAGTACG | 89 | NM_214201.1 |
|  | R: CGCCATTCACCTCACACTTCTCG |  |  |
| *IL-1β* | F: ACATGCTGAAGGCTCTCCAC | 170 | NM_214055.1 |
|  | R: CAGGGTGGGCGTGTTATCTT |  |  |
| *IL-6* | F: CGGATGCTTCCAATCTGGGT | 179 | NM_001252429.1 |
|  | R: TTCCCTTTTGCCTCAGGGTC |  |  |
| *IL-8* | F: TCCAAACTGGCTGTTGCCTTCTTG | 132 | M86923.1 |
|  | R: GGGGTGGAAAGGTGTGGAATGC |  |  |
| *IL-10* | F: CGGCCCAGTGAAGAGTTTCT | 151 | NM_214041.1 |
|  | R: TGCCTTCGGCATTACGTCTT |  |  |
| *IL-17* | F: CTCGTGAAGGCGGGAATCAT | 113 | NM_001005729.1 |
|  | R: GGTGTGCTCCGGTTCAAGAT |  |  |
| *IL-22* | F: GTGCTGTTCCCCAACTCTGA | 79 | XM_021091967.1 |
|  | R: GCTTTTTGCTGAGGCTGTCC |  |  |
| *TNF-α* | F: TCTCCTCCCTCCAGCCAATGT | 156 | AY572787.1 |
|  | R: CTGAAGAGGACCTGGGAGTAGA |  |  |
| *NF-kB* | F: TTCTGGACCGCTTGGGTAAC | 120 | NM_001048232.1 |
|  | R: CACCGTTGGGGTGGTTGATA |  |  |
| *Occludin* | F: CCTCCTCCCCTTTCGGACTA | 70 | NM_001163647.2 |
|  | R: TCACTTTCCCGTTGGACGAG |  |  |
| *ZO-1* | F: TCAAGGTCTGCCGAGACAAC | 140 | XM_021098896.1 |
|  | R: ATCACAGTGTGGTAAGCGCA |  |  |
| *GPR41* | F: ACTGGCTCTACTTCTCCGTGTACC | 133 | NM_001315601 |
|  | R: GTCAGGTTCAGCAGGAGCACATC |  |  |
| *GPR43* | F: CTCCTTAACTTCCTGGTGTGCTTCG | 80 | NM_001278758 |
|  | R: CATTTGGGGCTCTCCTTCGTGTG |  |  |

**Additional file 1: Table S6** Effects of RES supplementation on Alpha diversity of fecal microbiota in OSO-challenged weaned piglets^1^

| **Item** | **Fresh soybean oil** | | **Oxidized soybean oil** | | **SEM** | ***P*-value** | | |
| --- | --- | --- | --- | --- | --- | --- | --- | --- |
|  | **0 mg/kg**  **RES** | **300 mg/kg**  **RES** | **0 mg/kg**  **RES** | **300 mg/kg**  **RES** |  | **Oil** | **Res** | **Oil × Res** |
| OTU | 569.43 | 499.29 | 504.71 | 457.00 | 28.13 | 0.360 | 0.315 | 0.847 |
| Shannon | 7.44 | 6.62 | 7.29 | 6.83 | 0.18 | 0.949 | 0.093 | 0.629 |
| Simpson | 0.99 | 0.93 | 0.99 | 0.97 | 0.012 | 0.443 | 0.206 | 0.443 |
| Chao1 | 577.63 | 508.04 | 512.85 | 462.42 | 38.98 | 0.360 | 0.320 | 0.873 |

^1^ All of the values are expressed as the means and pooled SEM, *n* = 7, Means without a common letter differ (*P* < 0.05)

**Additional file 1: Table S7** Pearson correlation analyses between key genera and growth performance, nutrient apparent digestibility, intestinal histomorphology, antioxygenic properties, inflammatory cytokines, intestinal enzymatic activities and SCAFs

| **Genus** | **Performance** | **Correlation coefficient** | ***P*-value** |
| --- | --- | --- | --- |
| *Prevotella_1* | Chymotrypsin | -0.834 | 0.166 |
| *Prevotellaceae_UCG-003* | VH | -0.831 | 0.169 |
| *Prevotella_1* | CD (Jejunum) | -0.822 | 0.178 |
| *Atopobiaceae_unclassified* | VH | 0.814 | 0.186 |
| *Prevotella_1* | DAO | 0.805 | 0.195 |
| *Mollicutes_RF39_unclassified* | VCR | 0.790 | 0.210 |
| *Prevotella_1* | IL-8 | 0.771 | 0.229 |
| *Prevotella_1* | D-lactic acid | 0.760 | 0.240 |
| *Mollicutes_RF39_unclassified* | Acetic acid | 0.757 | 0.243 |
| *Prevotella_1* | TNF-α | 0.749 | 0.251 |
| *Prevotellaceae_UCG-003* | IL-1β | 0.749 | 0.251 |
| *Prevotellaceae_UCG-003* | IL-8 | 0.746 | 0.254 |
| *Mollicutes_RF39_unclassified* | D-lactic acid | -0.744 | 0.256 |
| *Atopobiaceae_unclassified* | H_2_O_2_ | -0.742 | 0.258 |
| *Prevotellaceae_UCG-003* | TNF-α | 0.737 | 0.263 |
| *Prevotella_1* | VCR | -0.725 | 0.275 |
| *Prevotella_1* | IL-1β | 0.717 | 0.283 |
| *Parabacteroides* | CD (Jejunum) | -0.714 | 0.286 |
| *Atopobiaceae_unclassified* | CD (Jejunum) | 0.707 | 0.293 |
| *Clostridium_sensu_stricto_6* | H_2_O_2_ | -0.692 | 0.308 |
| *Clostridium_sensu_stricto_6* | T-SOD | -0.685 | 0.315 |
| *Mollicutes_RF39_unclassified* | Chymotrypsin | 0.679 | 0.321 |
| *Prevotellaceae_UCG-003* | Chymotrypsin | -0.672 | 0.328 |
| *Mollicutes_RF39_unclassified* | Propionic acid | -0.666 | 0.334 |
| *Parabacteroides* | Acetic acid | 0.659 | 0.341 |
| *Prevotella_1* | Trypsin | -0.658 | 0.342 |
| *Mollicutes_RF39_unclassified* | Butyric acid | 0.647 | 0.353 |
| *Parabacteroides* | Butyric acid | 0.639 | 0.361 |
| *Prevotella_1* | Lipase | -0.629 | 0.371 |
| *Parabacteroides* | CD (Colon) | 0.628 | 0.372 |
| *Prevotellaceae_UCG-003* | DAO | 0.622 | 0.378 |
| *Atopobiaceae_unclassified* | α- amylase | 0.610 | 0.390 |
| *Prevotellaceae_UCG-003* | T-SOD | -0.607 | 0.393 |
| *Prevotellaceae_UCG-003* | D-lactic acid | 0.607 | 0.393 |
| *Prevotella_1* | Acetic acid | -0.589 | 0.411 |
| *Atopobiaceae_unclassified* | Propionic acid | 0.584 | 0.416 |
| *Clostridium_sensu_stricto_6* | IL-6 | -0.573 | 0.427 |
| *Parabacteroides* | α- amylase | -0.572 | 0.428 |
| *Prevotella_1* | CD (Colon) | -0.568 | 0.432 |
| *Prevotella_1* | IL-6 | 0.556 | 0.444 |
| *Parabacteroides* | Lipase | 0.549 | 0.451 |
| *Prevotellaceae_UCG-003* | IL-6 | 0.547 | 0.453 |
| *Prevotella_1* | Butyric acid | -0.530 | 0.470 |
| *Prevotellaceae_UCG-003* | VCR | -0.521 | 0.479 |
| *Parabacteroides* | VCR | 0.520 | 0.480 |
| *Atopobiaceae_unclassified* | Trypsin | 0.514 | 0.486 |
| *Prevotella_1* | H_2_O_2_ | 0.503 | 0.497 |
| *Mollicutes_RF39_unclassified* | T-SOD | -0.494 | 0.506 |
| *Parabacteroides* | IL-1β | 0.487 | 0.513 |
| *Clostridium_sensu_stricto_6* | *D*-lactic acid | 0.484 | 0.516 |
| *Prevotellaceae_UCG-003* | Trypsin | -0.470 | 0.530 |
| *Clostridium_sensu_stricto_6* | Trypsin | 0.461 | 0.539 |
| *Clostridium_sensu_stricto_6* | EE | -0.455 | 0.545 |
| *Prevotellaceae_UCG-003* | Lipase | -0.440 | 0.560 |
| *Prevotella_1* | Propionic acid | 0.427 | 0.573 |
| *Mollicutes_RF39_unclassified* | DAO | -0.426 | 0.574 |
| *Clostridium_sensu_stricto_6* | Lipase | -0.425 | 0.575 |
| *Mollicutes_RF39_unclassified* | IL-1β | 0.422 | 0.578 |
| *Mollicutes_RF39_unclassified* | CD (Jejunum) | -0.410 | 0.590 |
| *Atopobiaceae_unclassified* | DAO | -0.407 | 0.593 |
| *Mollicutes_RF39_unclassified* | IL-6 | 0.401 | 0.599 |
| *Prevotella_1* | T-SOD | -0.376 | 0.624 |
| *Parabacteroides* | D-lactic acid | -0.375 | 0.625 |
| *Prevotellaceae_UCG-003* | H_2_O_2_ | 0.375 | 0.625 |
| *Prevotellaceae_UCG-003* | Propionic acid | 0.372 | 0.628 |
| *Atopobiaceae_unclassified* | CD (Colon) | -0.365 | 0.635 |
| *Clostridium_sensu_stricto_6* | CD (Colon) | -0.363 | 0.637 |
| *Clostridium_sensu_stricto_6* | TNF-α | -0.362 | 0.638 |
| *Prevotellaceae_UCG-003* | CD (Colon) | -0.360 | 0.640 |
| *Clostridium_sensu_stricto_6* | IL-8 | -0.350 | 0.650 |
| *Parabacteroides* | TNF-α | 0.349 | 0.651 |
| *Parabacteroides* | Chymotrypsin | 0.344 | 0.656 |
| *Clostridium_sensu_stricto_6* | Chymotrypsin | -0.342 | 0.658 |
| *Prevotellaceae_UCG-003* | Acetic acid | -0.337 | 0.663 |
| *Atopobiaceae_unclassified* | EE | 0.337 | 0.663 |
| *Clostridium_sensu_stricto_6* | Butyric acid | 0.334 | 0.666 |
| *Parabacteroides* | IL-6 | 0.331 | 0.669 |
| *Clostridium_sensu_stricto_6* | α- amylase | -0.330 | 0.670 |
| *Atopobiaceae_unclassified* | Lipase | -0.328 | 0.672 |
| *Clostridium_sensu_stricto_6* | IL-1β | -0.321 | 0.679 |
| *Parabacteroides* | Trypsin | 0.315 | 0.685 |
| *Parabacteroides* | IL-8 | 0.310 | 0.690 |
| *Mollicutes_RF39_unclassified* | Trypsin | 0.310 | 0.690 |
| *Parabacteroides* | DAO | -0.299 | 0.701 |
| *Mollicutes_RF39_unclassified* | TNF-α | 0.293 | 0.707 |
| *Parabacteroides* | VH | -0.283 | 0.717 |
| *Prevotellaceae_UCG-003* | Butyric acid | -0.277 | 0.723 |
| *Clostridium_sensu_stricto_6* | CD (Jejunum) | -0.254 | 0.746 |
| *Mollicutes_RF39_unclassified* | IL-8 | 0.244 | 0.756 |
| *Clostridium_sensu_stricto_6* | VCR | -0.242 | 0.758 |
| *Parabacteroides* | Propionic acid | -0.239 | 0.761 |
| *Parabacteroides* | EE | -0.235 | 0.765 |
| *Mollicutes_RF39_unclassified* | α- amylase | -0.218 | 0.782 |
| *Atopobiaceae_unclassified* | T-SOD | 0.215 | 0.785 |
| *Clostridium_sensu_stricto_6* | VH | 0.211 | 0.789 |
| *Clostridium_sensu_stricto_6* | DAO | -0.203 | 0.797 |
| *Atopobiaceae_unclassified* | D-lactic acid | 0.193 | 0.807 |
| *Mollicutes_RF39_unclassified* | EE | 0.180 | 0.820 |
| *Mollicutes_RF39_unclassified* | VH | -0.142 | 0.858 |
| *Atopobiaceae_unclassified* | Butyric acid | 0.142 | 0.858 |
| *Clostridium_sensu_stricto_6* | Acetic acid | 0.131 | 0.869 |
| *Atopobiaceae_unclassified* | VCR | -0.118 | 0.882 |
| *Parabacteroides* | H_2_O_2_ | -0.101 | 0.899 |
| *Mollicutes_RF39_unclassified* | H_2_O_2_ | 0.028 | 0.972 |
| *Atopobiaceae_unclassified* | Chymotrypsin | -0.024 | 0.976 |
| *Atopobiaceae_unclassified* | Acetic acid | 0.005 | 0.995 |
